# Assessing methods to quantitatively validate TGFβ-dependent autophagy

**DOI:** 10.1242/bio.055103

**Published:** 2020-11-26

**Authors:** Charles B. Trelford, Gianni M. Di Guglielmo

**Affiliations:** Schulich School of Medicine and Dentistry, Western University, Department of Physiology and Pharmacology, London, Ontario, Canada N6A 5B7

**Keywords:** Autophagic flux, Autophagy, Microtubule-associated protein light chain 3B (LC3B), Non-small cell lung cancer (NSCLC) and Transforming growth factor beta (TGFβ)

## Abstract

Transforming growth factor beta (TGFβ) promotes tumorigenesis by suppressing immune surveillance and inducing epithelial to mesenchymal transition (EMT). TGFβ may augment tumorigenesis by activating autophagy, which protects cancer cells from chemotherapy and promotes invasive and anti-apoptotic properties. Here, we assess how TGFβ1 modulates autophagy related (*ATG*) gene expression and ATG protein levels. We also assessed microtubule-associated protein light chain 3 (LC3) lipidation, LC3 puncta formation and autophagosome-lysosome co-localization in non-small cell lung cancer (NSCLC) cell lines. These experimental approaches were validated using pharmacological autophagy inhibitors (chloroquine and spautin-1) and an autophagy activator (MG132). We found that TGFβ1, chloroquine and MG132 had little effect on ATG protein levels but increased LC3 lipidation, LC3 puncta formation and autophagosome-lysosome co-localization. Since similar outcomes were observed using chloroquine and MG132, we concluded that several techniques employed to assess TGFβ-dependent autophagy may not differentiate between the activation of autophagy versus lysosomal inhibition. Thus, NSCLC cell lines stably expressing a GFP-LC3-RFP-LC3ΔG autophagic flux probe were used to assess TGFβ-mediated autophagy. Using this approach, we observed that TGFβ, MG132 and serum starvation increased autophagic flux, whereas chloroquine and spautin-1 decreased autophagic flux. Finally, we demonstrated that ATG5 and ATG7 are critical for TGFβ-dependent autophagy in NSCLC cells. The application of this model will fuel future experiments to characterize TGFβ-dependent autophagy, which is necessary to understand the molecular processes that link, TGFβ, autophagy and tumorigenesis.

## Abstract (precis)

**Summary:** Using various tools including western blotting and immunofluorescence microscopy, it was observed that autophagic flux probes are the most robust tools to quantitatively assess cellular autophagy.

## INTRODUCTION

Transforming growth factor beta (TGFβ) is a pleotropic cytokine that regulates cell growth, migration, apoptosis and proliferation (McLean and Di Guglielmo, 2014). In non-cancerous cells, TGFβ impedes tumor formation; however, in tumor cells, TGFβ enhances tumorigenesis by enabling epithelial to mesenchymal transition (EMT), dampening immune surveillance and promoting angiogenesis (Batlle and Massagué, 2019). Additionally, TGFβ may promote tumorigenesis by activating macroautophagy (Ding et al., 2010; Kiyono et al., 2009), hereafter referred to as autophagy, which is a catabolic process facilitated by the formation of double membrane vesicles called autophagosomes that sequester and transport cellular cargo to lysosomes (Mathew et al., 2007; Yim and Mizushima, 2020).

Briefly, autophagy is initiated by recruiting the UNC-51-like kinase complex to the rough endoplasmic reticulum (Kaur and Debnath, 2015). A class III phosphoinositide 3 kinase complex consisting of a vacuolar protein sorting 34 phosphoinositide 3 kinase and beclin1 (BECN1) binds to the UNC-51-like kinase (ULK) complex, which initiates the formation of an isolated lipid barrier known as a phagophore (Tanida, 2011; Bernard and Klionsky, 2013). Phagophore elongation is facilitated by two ubiquitin-like conjugation reactions (Walczak and Martens, 2013; Takahashi et al., 2019). The first reaction is initiated when autophagy related (ATG) protein 4 (ATG4) cleaves the microtubule-associated protein light chain 3B (LC3B), which produces LC3B-I. LC3B-I is activated by ATG7 and conjugated to phosphatidylethanolamine by ATG3 to form LC3B-II, which is referred to as LC3B lipidation (Glick et al., 2010). The second ubiquitin-like conjugation reaction produces an ATG5-ATG12-ATG16L1 complex that encloses phagophores around cellular cargo by adding lipids and LC3B-II to phagophore membranes (Walczak and Martens, 2013; Chen et al., 2012). Eventually, the phagophore matures to create an autophagosome that migrate via microtubules toward lysosomes (Mackeh et al., 2013). Finally, autophagosome-lysosome fusion creates an autolysosome, which is the location of hydrolase-dependent degradation of autophagosomes and enclosed cellular cargo (Levine et al., 2011; Xu and Ren, 2015).

Like TGFβ, autophagy has a complex relationship with cancer (Vera-Ramirez et al., 2018; Mowers et al., 2017). For instance, using lysosomal enzymes to recycle unwanted, superfluous and aggregated proteins and eliminate damaged organelles, autophagy regulates iron, carbohydrate, fatty acid, amino acid and cholesterol homeostasis (Kaur and Debnath, 2015; Gatica et al., 2018). As such, autophagy protects cells from tumorigenesis, which was verified by spontaneous tumor formation in *ATG* gene knockout models (Takamura et al., 2011). Paradoxically, in cancer cells, autophagy has been linked to EMT, anoikis resistance, stem cell phenotypes, quiescent phenotypes, cell migration and resistance to cancer therapies, which augment tumorigenesis (Mathew et al., 2007; Eskelinen, 2011). For these reasons, there is a growing interest in generating autophagy inhibitors to impede the tumor promoting properties of autophagy (Rebecca and Amaravadi, 2016). For example, autophagy inhibition has been shown to attenuate TGFβ-dependent EMT (Alizadeh et al., 2018; Qiang and He, 2014). Therefore, autophagy has become an attractive therapeutic target for tumors expressing elevated concentrations of TGFβ (Wu et al., 2018; Ghavami et al., 2015).

The literature suggests that TGFβ upregulates the expression of *ATG* genes (Xu et al., 2012), increases the levels of ATG proteins (Fu et al., 2014), induces LC3 puncta formation (Ding et al., 2010), promotes LC3-lysosome co-localization and increases the number of autophagosomes (Alizadeh et al., 2018). However, several experimental techniques utilized to investigate TGFβ-dependent autophagy have caveats that may result in varying interpretations (Klionsky et al., 2016). For this reason, highlighting potential technical pitfalls in the investigation of TGFβ-dependent autophagy and using strategies designed to more accurately interpret the impact of TGFβ on autophagy will be helpful to the field of TGFβ biology. By using non-small cell lung cancer (NSCLC) cells, we examined several experimental approaches to quantitatively and reliably investigate TGFβ-dependent autophagy (Kaizuka et al., 2016).

## RESULTS

### TGFβ1 has little effect on the expression of ATG genes in A549 NSCLC cell lines

The purpose of this work was to explore different techniques to provide quantitative evidence that TGFβ1 induces autophagy in NSCLC cells. In order to examine how TGFβ1 regulated autophagy, we first utilized microarray analysis to determine the effect of TGFβ1 on the expression of *ATG* genes in A549 cells (Table 1). A549 cells were treated with 250 pM TGFβ1 for 0 h (control) or 1 h, which was followed by an 8 h or 24 h washout. We observed that TGFβ1 elicited only a modest change in the expression of *ATG* genes. Indeed, there was a small increase in genes that encode ATG4D, ATG9A, ATG16L1, GABA Type A Receptor-Associated Protein L1, GABA Type A Receptor-Associated Protein L3 and microtubule-associated protein light chain 3A; and a minor decrease in the expression of *ATG3*. The presence and activity of TGFβ1 was verified by the increase in the expression of *CDH2*, a mesenchymal marker, and a decrease in the expression of *CDH1*, an epithelial marker, which are known targets of TGFβ1 signaling (Table 1) (Ganesan et al., 2016; Karlsson et al., 2017). Thus, although TGFβ1 had a modest effect on the expression of some autophagy-related genes, it had little effect on the expression of the majority of *ATG* genes in A549 cells.

**Table 1.**
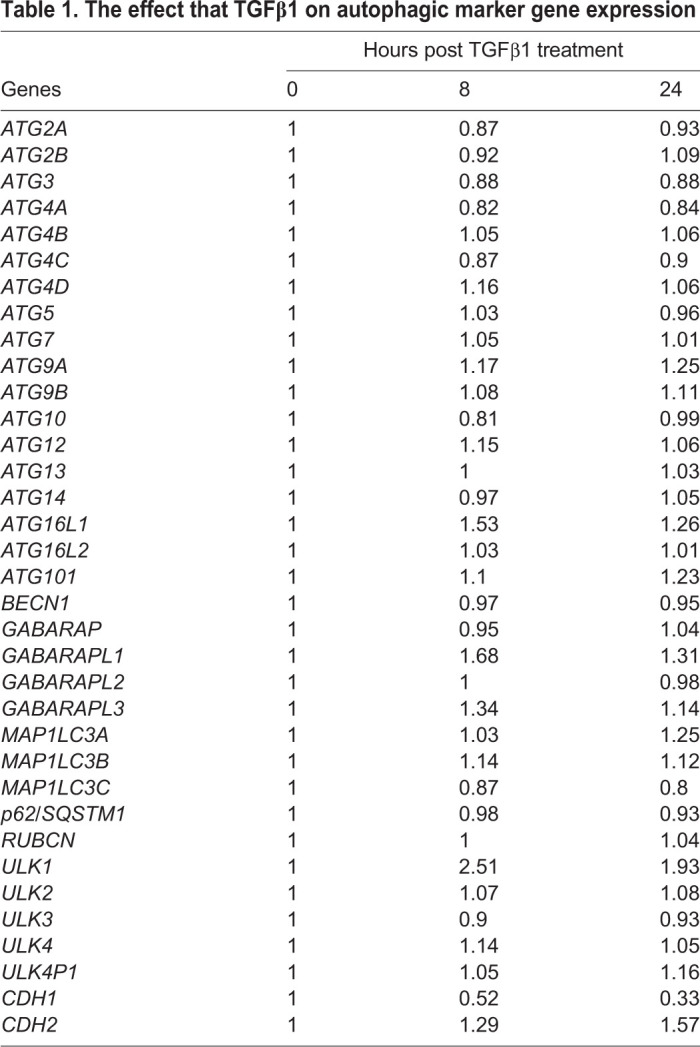
**The effect that TGFβ1 on autophagic marker gene expression**

### TGFβ1 induces LC3B lipidation but does not increase ATG protein levels in NSCLC cell lines

**Fig. 1.**
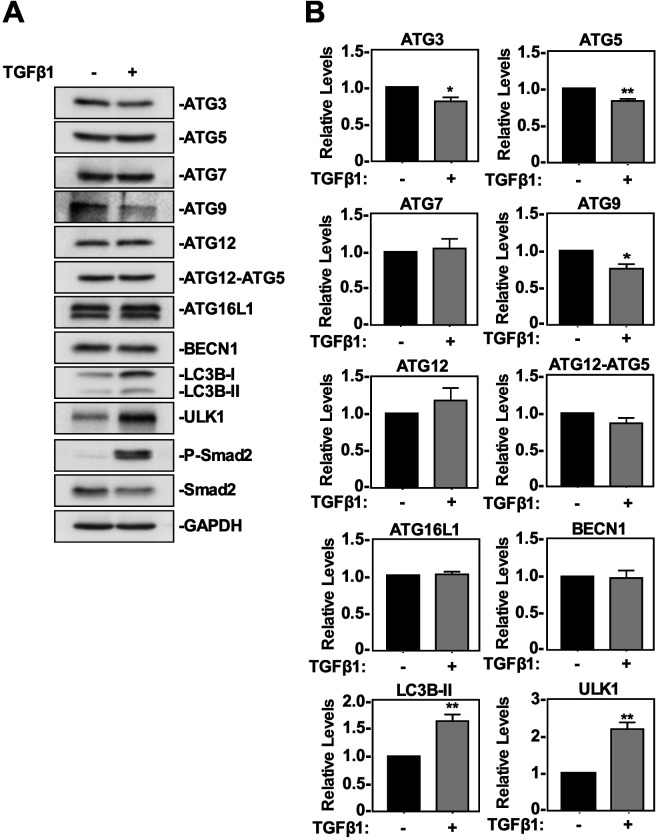
**The effect of TGFβ1 on ATG protein levels and LC3B lipidation in A549 cells.** (A) A549 cells were treated with 250 pM TGFβ1 for 24 h. Cells were lysed and subjected to SDS-PAGE and immunoblotting anti-ATG3, anti-ATG5, anti-ATG7, anti-ATG9, anti-ATG12, anti-ATG12-ATG5 complex, anti-ATG16L1, anti-BECN1, anti-ULK1, anti-LC3B, anti-P-Smad2, anti-Smad2 and anti-GAPDH (loading control) antibodies. (B) The steady state levels of ATG3, ATG5, ATG7, ATG9, ATG12, ATG12-ATG5, ATG16L1, BECN1, ULK1, and LC3B were quantitated using QuantityOne software and graphed (*n*=3±s.e.m.). Significance is indicated as *=*P*<0.05, **P<0.01 and ****=*P*<0.0001.

We next assessed the effect of TGFβ1 on the steady state levels of several ATG proteins that facilitate or regulate autophagy. A549 cells and H1299 cells were treated with 250 pM TGFβ1 for 24 h prior to lysis and immunoblotted for autophagy related proteins whose genes were found to be induced (ATG9A, ATG16L1 and ULK1), reduced (ATG3) or unchanged (ATG5, ATG7, ATG12 and ATG12/5 complex, Beclin 1 and LC3B) in Table 1 (Fig. 1). Furthermore, we also immunoblotted for phospho-Smad2 (P-Smad2), Smad2, and GAPDH (loading control). P-Smad2 verified the presence and activity of TGFβ1 in both cell lines. In A549 cells, TGFβ1 had no significant impact on the protein levels of ATG7, BECN1, ATG12 or ATG12-ATG5 complex formation. Interestingly, TGFβ decreased the protein levels of ATG3, ATG5 and ATG9, whereas it increased ULK1 and LC3B-II protein levels (Fig. 1). In H1299 cells, TGFβ1 had no significant impact on the protein levels of BECN1, ATG3, ATG5, ATG12 or ATG12-ATG5 complex formation. However, in this cell line, TGFβ1 significantly decreased ATG7 and ATG9 protein levels and increased ULK1 and LC3B-II protein levels (Fig. S1). Therefore, after assessing the impact that TGFβ1 had on steady state ATG proteins, we found that the levels of ULK1 and LC3B were consistent indicators of TGFβ1-induced autophagy in both NSCLC cell lines.

### Using LC3B lipidation and ATG protein levels as readouts for TGFβ1-induced autophagy

Although LC3-II protein levels are considered to be proportional to the amount of autophagosomes, their utility as an indicator for autophagy remains unresolved (Klionsky et al., 2016). For this reason, we investigated if using LC3 lipidation would be useful to draw conclusions regarding TGFβ1-induced autophagy. First, we analyzed how known inhibitors and activators of autophagy impacted LC3 lipidation. The pharmacological inhibitors of autophagy selected for the study were chloroquine (Redmann et al., 2017) and spautin-1 (Shao et al., 2014) whereas MG132, a proteasomal inhibitor, functioned as an activator of autophagy (Bao et al., 2016). For each compound, we determined the optimal dose and treatment duration that would have a significant effect on LC3B-II protein levels but would not affect cell viability, as assessed by MTT assays in both A549 cells (Fig. S2) and H1299 cells (Fig. S3). The doses and treatment durations that had low cell mortality (50 µM chloroquine, 10 µM spautin-1 and 5 µM MG132 for 24 h) were selected as the treatment regimen for each pharmacological autophagy modulator (Figs S2 and S3). In both cell lines, spautin-1 decreased steady state LC3B-II protein levels, whereas chloroquine and MG132 increased steady state LC3B-II protein levels. Based on our observations that both chloroquine (an inhibitor of autophagy) and MG132 (an activator of autophagy) increased LC3B-II protein levels, the TGFβ1-dependent increase in LC3B lipidation was insufficient to conclude that TGFβ1 activated autophagy.

**Fig. 2.**
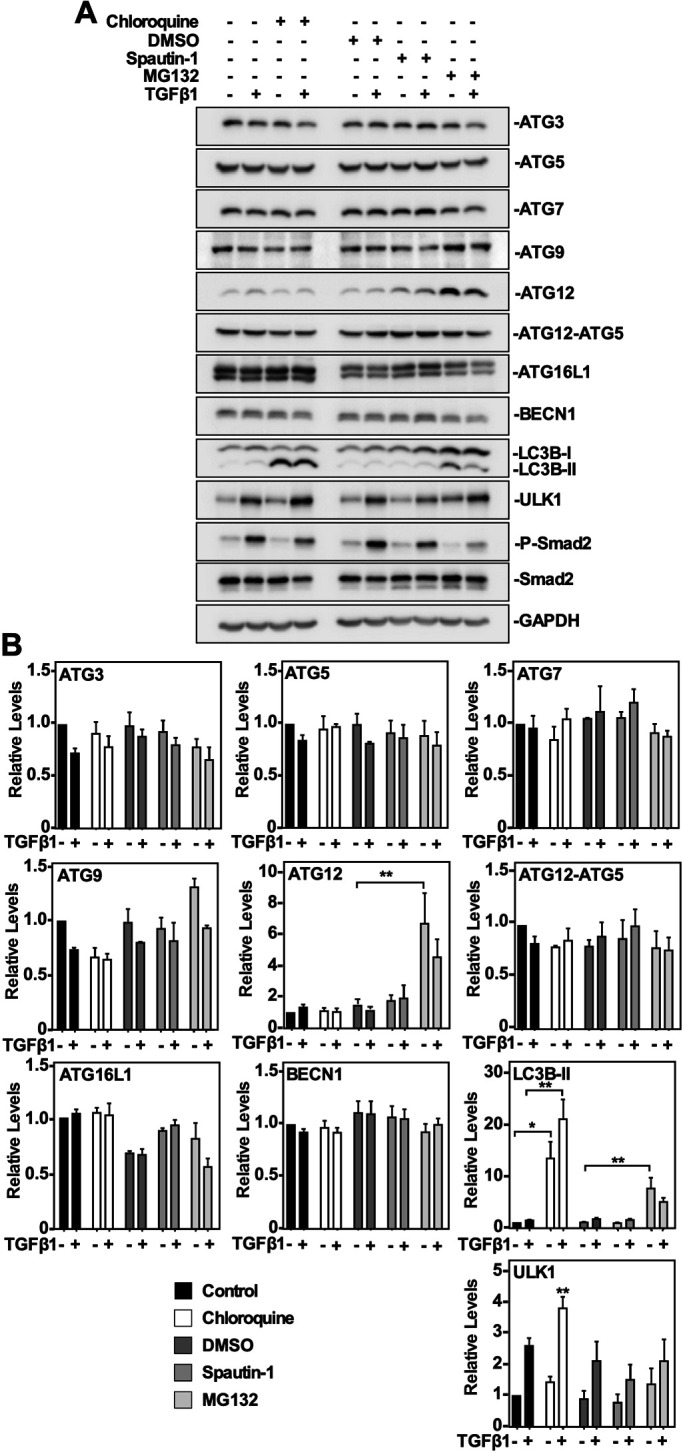
**The effect of chloroquine, spautin-1 and MG132 on ATG protein levels in A549 cells.** (A) A549 cells were treated with 50 μM chloroquine, 10 μM spautin-1 or 5 μM MG132 in the presence or absence of 250 pM TGFβ1 for 24 h. Cells were lysed and subjected to SDS-PAGE and immunoblotting anti-ATG3, anti-ATG5, anti-ATG7, anti-ATG9, anti-ATG12, anti-ATG12-ATG5 complex, anti-ATG16L1, anti-BECN1, anti-ULK1, anti-LC3B, anti-P-Smad2, anti-Smad2 and anti-GAPDH (loading control) antibodies. (B) The steady state levels of ATG3, ATG5, ATG7, ATG9, ATG12, ATG12-ATG5, ATG16L1, BECN1, ULK1, and LC3B were quantitated using QuantityOne software and graphed (*n*=3, mean±s.e.m.). Significance is indicated as *=*P*<0.05, **P<0.01 and ****=*P*<0.0001.

As shown above, TGFβ1 had consistent effects on three of the ten ATG-related protein levels (ATG9, ULK1 and LC3B) in A549 cells and H1299 cells. To study this further, we treated A549 cells and H1299 cells with 50 µM chloroquine, 10 µM spautin-1 or 10 µM MG132 in the presence and absence of 250 pM TGFβ1 for 24 h. The cells were lysed, and immunoblotted for ATG3, ATG5, ATG7, ATG9, ATG12, ATG12-ATG5 complex formation, BECN1, ULK1, and LC3B-II (Fig. 2). Once again, P-Smad2 and Smad2 levels were assessed to confirm TGFβ1 activity. In both cell lines, there were no significant changes in the protein levels of ATG3, ATG5, ATG7, ATG9, BECN1, ULK1, ATG16L1 or ATG12-ATG5 complex formation; MG132 increased the steady state of ATG12 and LC3B-II protein levels and chloroquine significantly increased steady state LC3B-II protein levels (Fig. 2; Fig. S4). These results suggested that using steady state ATG protein levels to assess TGFβ1-induced autophagy is inconclusive due to the fact that ATG protein levels are relatively stable despite chloroquine, spautin-1 and MG132 treatment.

### Assessing autophagy using LC3 puncta formation and LC3-lysosome co-localization in A549 cells

**Fig. 3.**
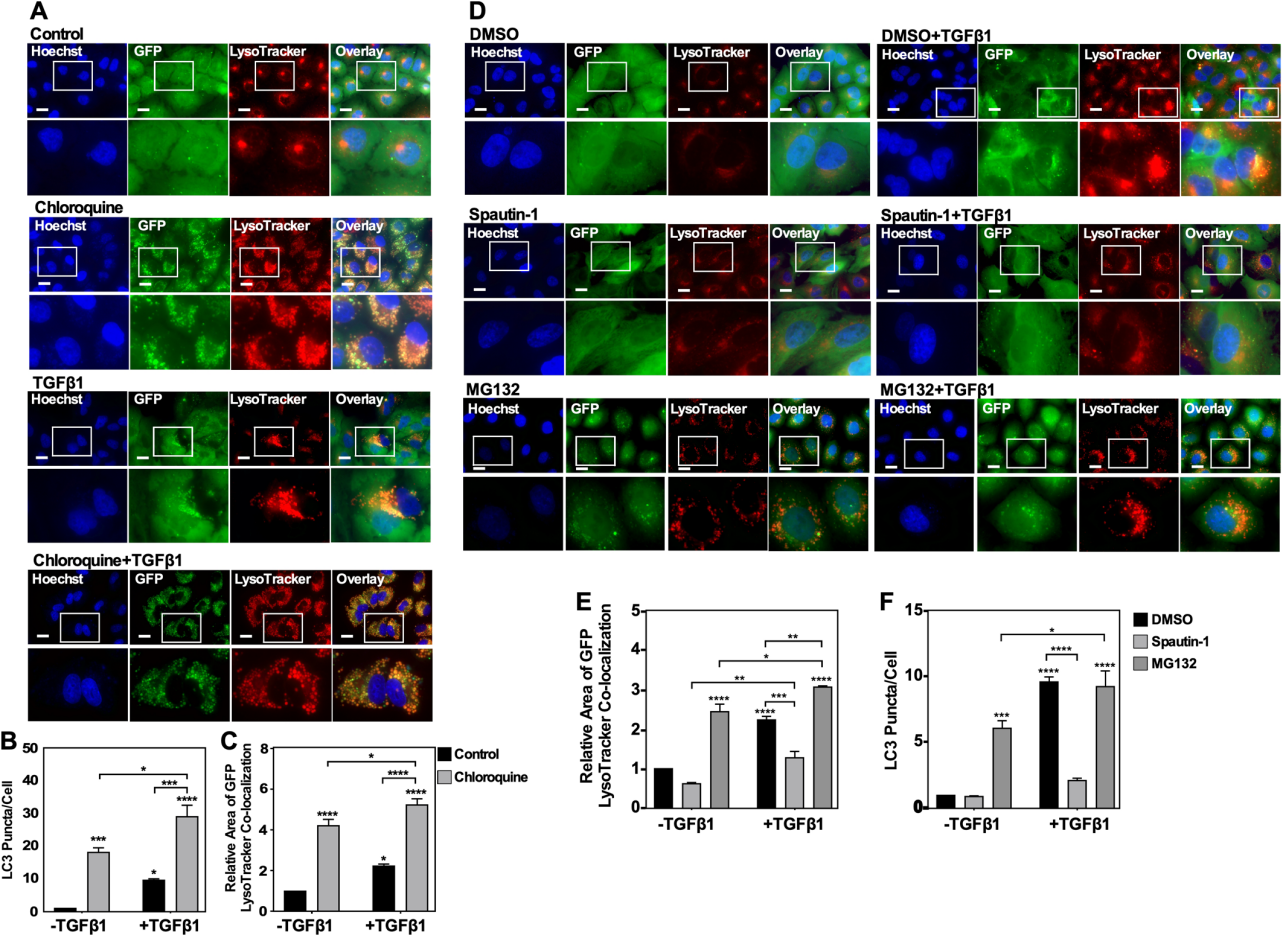
**The effect of pharmacological modulation of autophagy and TGFβ1 on LC3 puncta formation and autophagosome-lysosome co-localization.** (A) A549 cells transfected with a cDNA GFP-LC3-RFP-LC3ΔG vector were treated with 50 μM chloroquine in the presence or absence of 250 pM TGFβ1. LysoTracker Deep Red (red) and Hoechst stain (blue) were added 2 h and 10 min, respectively, prior to imaging. Images were obtained with a 63x objective using an Olympus IX 81 inverted fluorescence microscope. Scale bars: 10 μm. (B) Cells and number of puncta/cell were counted using ImageJ version 2.0 software. The data were graphed from three independent experiments (mean±s.e.m.). Significance is indicated as *=*P*<0.05, ***P<0.001 and ****=*P*<0.0001. (C) ImageJ version 2.0 was used to quantify the number of yellow pixels per cell area for chloroquine and no treatment. The data were graphed from three independent experiments (mean±s.e.m.). Significance is indicated as *=*P*<0.05 and ****=*P*<0.0001. (D) A549 cells transfected with a cDNA GFP-LC3-RFP-LC3ΔG vector were treated with 10 μM spautin-1, 10 μM MG132, or DMSO (vehicle control) in the presence or absence of 250 pM TGFβ1. LysoTracker Deep Red (red) and Hoechst stain (blue) were added 2 h and 10 min, respectively, prior to imaging. Images were obtained with a 63x objective using an Olympus IX 81 inverted fluorescence microscope. Scale bars: 10 μm. (E) ImageJ version 2.0 was used to count the number of cells and puncta per image for all treatments. The data were graphed from three independent experiments (mean±s.e.m.). Significance is indicated as *=*P*<0.05, **=*P*<0.01 ***P<0.001 and ****=*P*<0.0001. (F) ImageJ version 2.0 was used to quantify the number of yellow pixels per cell area for all treatments. The data were graphed from three independent experiments (mean±s.e.m.). Significance is indicated as *=*P*<0.05, ***=*P*<0.001 and ****=*P*<0.0001.

We next utilized A549 cells stably expressing GFP-labelled LC3 protein to determine if TGFβ1 increased GFP-LC3 puncta formation or GFP-LC3-lysosome co-localization. Although some GFP is quenched within the lysosomal lumen (Klionsky et al., 2016), GFP-LC3-lysosome co-localization was detected and fluctuated in response to treatment. Briefly, A549 cells were treated with 50 µM chloroquine, 10 µM spautin-1 or 10 µM MG132 in the presence and absence of 250 pM TGFβ1 for 24 h prior to LysoTracker Deep Red incubation to identify lysosomes (Fig. 3). After examining the images obtained from untreated or chloroquine-treated cells, we observed that 24 h of TGFβ1 increased GFP-LC3 puncta formation (Fig. 3B) and GFP-LC3-lysosome co-localization relative to untreated cells (Fig. 3C). This is consistent with TGFβ stimulating diffuse GFP-LC3 to target to lysosomes. We next examined the images containing cells that were treated with vehicle (DMSO), spautin-1 or MG132 (Fig. 3D), and observed that MG132 increased GFP-LC3 puncta formation and spautin-1 and TGFβ1 decreased GFP-LC3 puncta formation compared to the DMSO and TGFβ1 treatment, respectively (Fig. 3E). Finally, MG132 increased GFP-LC3-II-lysosome co-localization relative to DMSO (control) treatment, whereas the combination of spautin-1 and TGFβ1 decreased GFP-LC3-II-lysosome co-localization compared to TGFβ1 treatment alone (Fig. 3F). Since these results suggested that both chloroquine and MG132 increased GFP-LC3 puncta formation and GFP-LC3-II-lysosome co-localization, we were unable to conclude that the TGFβ1-dependent increase of GFP-LC3 puncta formation or LC3-lysosome co-localization represented an induction of autophagy.

### TGFβ1 increases autophagic flux

**Fig. 4.**
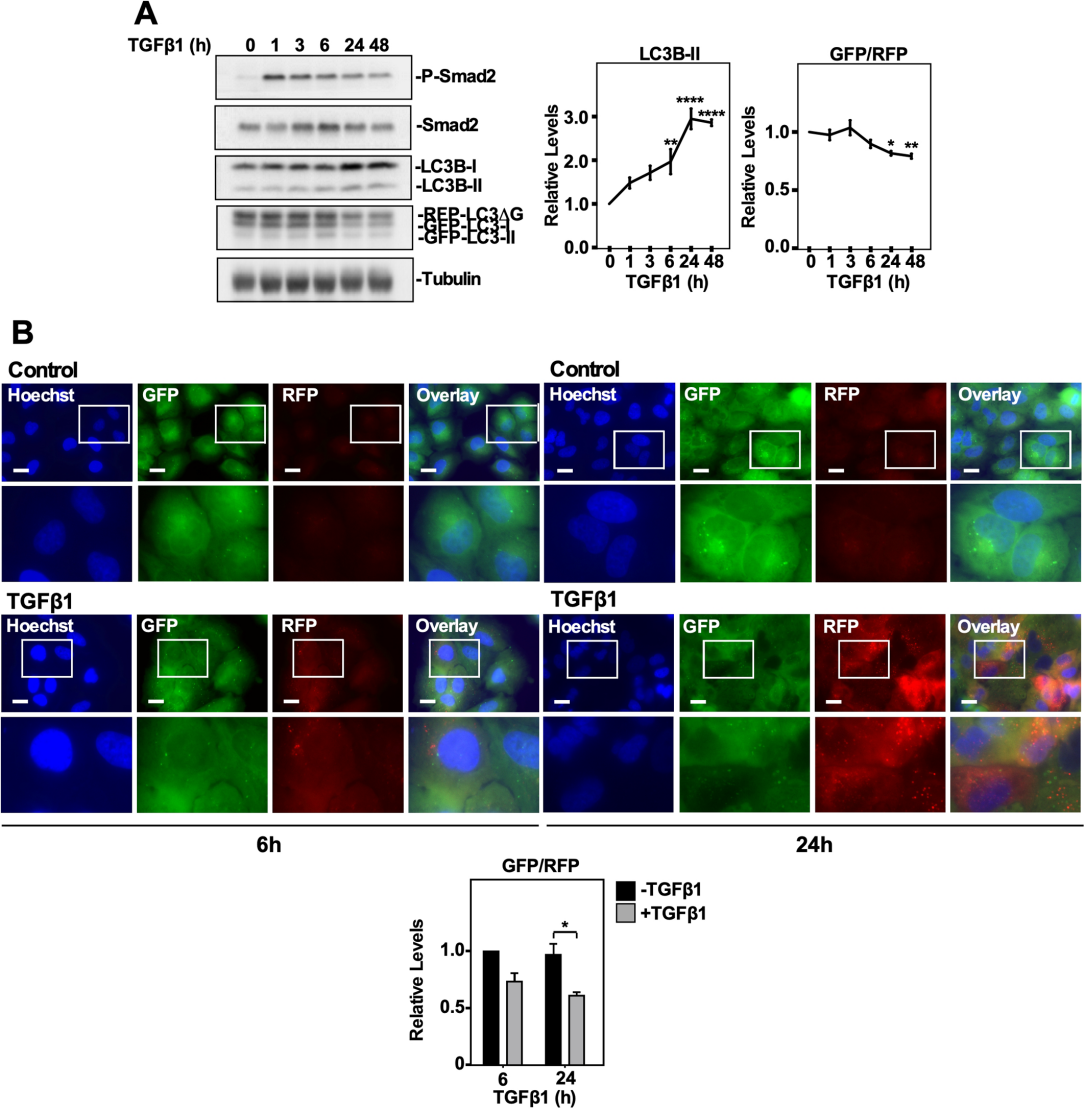
**Using a GFP-LC3-RFP-LC3ΔG probe to assess TGFβ1-dependent autophagy in A549 cells.** (A) A549 cells stably expressing a cDNA GFP-LC3-RFP-LC3ΔG construct were treated with 250 pM TGFβ1 for 0–48 h, lysed, subjected to SDS-PAGE and immunoblotted with anti-P-Smad2, anti-Smad2, anti-LC3B, anti-GAPDH and anti-tubulin antibodies. Quantitative analysis of steady state LC3B-II levels and the GFP/RFP ratio are shown graphically to the right of representative immunoblots. The data were graphed from three independent experiments (mean±s.e.m.). Significance is indicated as *=*P*<0.05, **P<0.01 and ****=*P*<0.0001. (B) A549 cells stably expressing a cDNA GFP-LC3-RFP-LC3ΔG construct were treated with 250 pM TGFβ1 for 6 or 24 h. Hoechst stain (blue) was added 10 min prior to imaging. Images were obtained with a 63× objective using an Olympus IX 81 inverted fluorescence microscope and ImageJ quantified the green and red pixel intensity. The GFP/RFP ratio is shown to the right of representative images. The data were graphed from three independent experiments (mean±s.e.m.). Significance is indicated as *=*P*<0.05. Scale bars: 10 μm.

Although we investigated the impact that TGFβ1 had on ATG gene expression, ATG protein levels, GFP-LC3 puncta formation and LC3-lysosome co-localization, the data did not consistently support the notion that TGFβ1 induced autophagy in NSCLC cell lines. We therefore next assessed the ability of TGFβ1 to alter autophagic flux in A549 cells and H1299 cells, using the GFP-LC3-RFP-LC3ΔG autophagic flux vector developed by the Mizushima laboratory (Kaizuka et al., 2016). Briefly, A549 cells and H1299 cells were generated to stably express the GFP-LC3-RFP-LC3ΔG reporter and the resulting GFP/RFP ratio was used to monitor autophagic flux, which was assessed via both immunoblotting and fluorescence microscopy (Fig. 4; Fig. S5). In A549 cells, quantitation of western blots indicated that 6–48 h of TGFβ1 incubation significantly increased steady state LC3B-II protein levels and 24 and 48 h of TGFβ1 significantly decreased the GFP/RFP ratio (Fig. 4A). Assessing autophagic flux in A549 cells using fluorescence microscopy revealed that 6 h of TGFβ1 did not impact the GFP/RFP ratio whereas 24 h of TGFβ1 significantly decreased the GFP/RFP ratio (Fig. 4B). In H1299 cells, quantitative analysis of the western blots indicated that steady state LC3B-II protein levels were significantly increased after 3 and 24 h of TGFβ1 incubation. Like A549 cells, 24 and 48 h of TGFβ1 significantly decreased the GFP/RFP ratio in H1299 cells (Fig. S5A). Furthermore, 24 h of TGFβ1 significantly decreased the GFP/RFP ratio in H1299 cells when it was assessed via fluorescence microscopy (Fig. S5B). Therefore, assessing the GFP/RFP ratio in GFP-LC3-RFP-LC3ΔG stably transfected cells suggested that TGFβ1 activated autophagy in both NSCLC cell lines.

### Verifying the GFP-LC3-RFP-LC3ΔG probe as an appropriate tool to assess autophagic flux

**Fig. 5.**
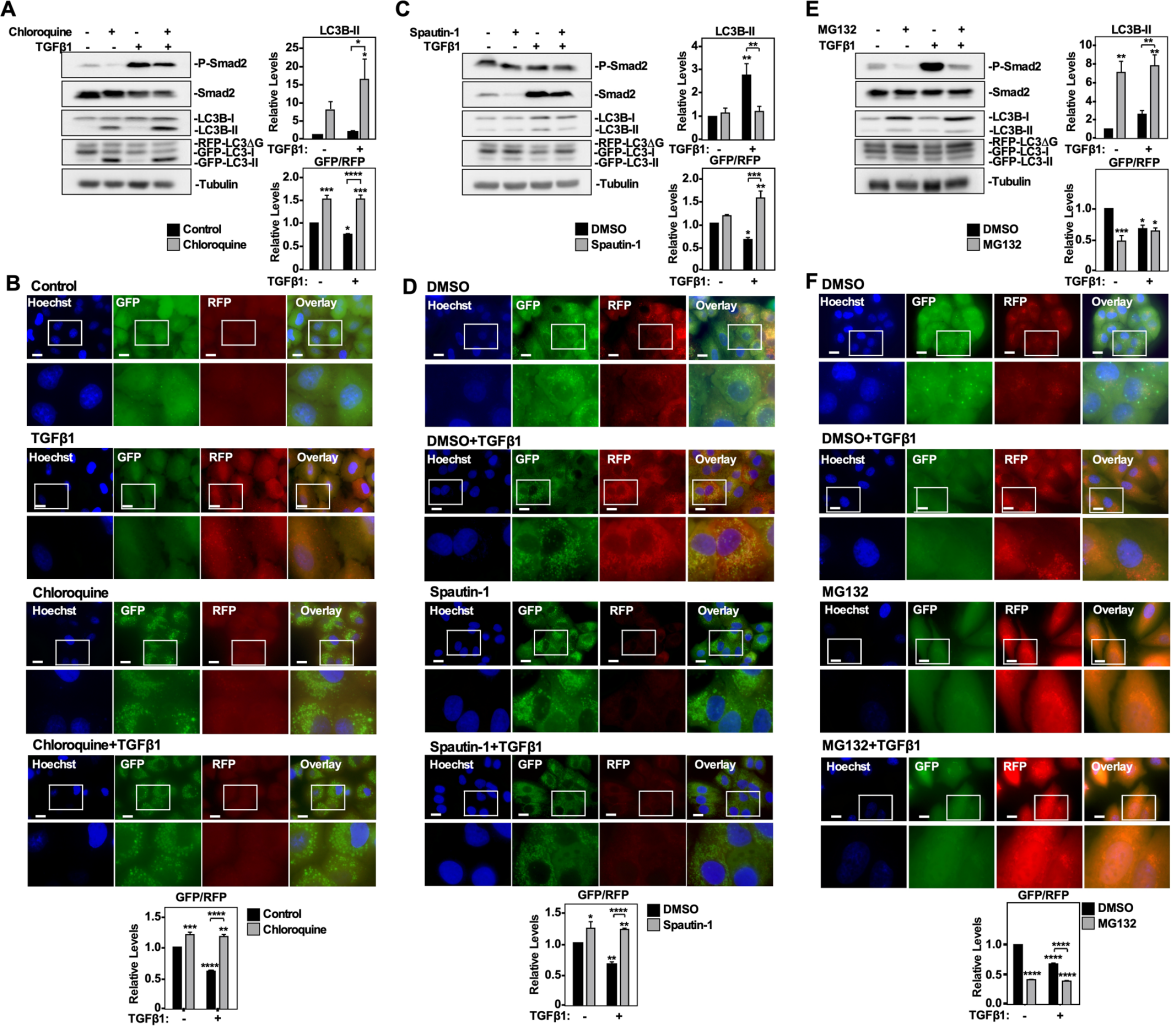
**The effect of chloroquine, spautin-1 and MG132 on autophagic flux in A549 cells.** (A) A549 cells stably expressing a cDNA GFP-LC3-RFP-LC3ΔG construct were treated with 50 μM chloroquine in the presence and absence of 250 pM TGFβ1 for 24 h, lysed, subjected to SDS-PAGE and immunoblotted with anti-P-Smad2, anti-Smad2, anti-LC3B, anti-GAPDH and anti-tubulin antibodies. Quantitative analysis of steady state LC3B-II levels and the GFP/RFP ratio are shown graphically to the right of representative immunoblots. The data were graphed from three independent experiments (mean±s.e.m.). Significance is indicated as *=*P*<0.05, ***P<0.001 and ****=*P*<0.0001. (B) A549 cells stably expressing a cDNA GFP-LC3-RFP-LC3ΔG construct were treated with 50 μM chloroquine in the presence and absence of 250 pM TGFβ1 for 6 or 24 h. Hoechst stain (blue) was added 10 min prior to imaging. Images were obtained with a 63x objective using an Olympus IX 81 inverted fluorescence microscope and ImageJ quantified the green and red pixel intensity. The GFP/RFP ratio is shown below representative images. The data were graphed from three independent experiments (mean±s.e.m.). Significance is indicated as **P<0.01, ***=*P*<0.001 and ****=*P*<0.0001. Scale bars: 10 μm. (C) A549 cells stably expressing a cDNA GFP-LC3-RFP-LC3ΔG construct were treated with 10 μM spautin-1 in the presence and absence of 250 pM TGFβ1 for 24 h, lysed, subjected to SDS-PAGE and immunoblotted with anti-P-Smad2, anti-Smad2, anti-LC3B, anti-GAPDH and anti-tubulin antibodies. Quantitative analysis of steady state LC3B-II levels and the GFP/RFP ratio are shown graphically to the right of representative immunoblots. The data were graphed from three independent experiments (mean±s.e.m.). Significance is indicated as *=*P*<0.05, **=*P*<0.01, ***P<0.001 and ****=*P*<0.0001. (D) A549 cells stably expressing a cDNA GFP-LC3-RFP-LC3ΔG construct were treated with 10 μM spautin-1 in the presence and absence of 250 pM TGFβ1 for 6 or 24 h. Hoechst stain (blue) was added 10 min prior to imaging. Images were obtained with a 63× objective using an Olympus IX 81 inverted fluorescence microscope and ImageJ quantified the green and red pixel intensity. The GFP/RFP ratio is shown below representative images. The data were graphed from three independent experiments (mean±s.e.m.). Significance is indicated as *=*P*<0.05, **P<0.01 and ****=*P*<0.0001. Scale bars: 10 μm. (E) A549 cells stably expressing a cDNA GFP-LC3-RFP-LC3ΔG construct were treated with 5 μM MG132 in the presence and absence of 250 pM TGFβ1 for 24 h, lysed, subjected to SDS-PAGE and immunoblotted with anti-P-Smad2, anti-Smad2, anti-LC3B, anti-GAPDH and anti-tubulin antibodies. Quantitative analysis of steady state LC3B-II levels and the GFP/RFP ratio are shown graphically to the right of representative immunoblots. The data were graphed from three independent experiments (mean±s.e.m.). Significance is indicated as *=*P*<0.05, **=*P*<0.01 and ***P<0.001. (F) A549 cells stably expressing a cDNA GFP-LC3-RFP-LC3ΔG construct were treated with 5 μM MG132 in the presence and absence of 250 pM TGFβ1 for 6 or 24 h. Hoechst stain (blue) was added 10 min prior to imaging. Images were obtained with a 63x objective using an Olympus IX 81 inverted fluorescence microscope and ImageJ quantified the green and red pixel intensity. The GFP/RFP ratio is shown below representative images. The data were graphed from three independent experiments (mean±s.e.m.). Significance is indicated as ****=*P*<0.0001. Scale bars: 10 μm.

Next, we assessed the accuracy of the GFP/RFP ratio obtained from cells stably transfected with a GFP-LC3-RFP-LC3ΔG vector at predicting autophagic flux. A549 cells and H1299 cells were treated with 50 µM chloroquine, 10 µM spautin-1 or 10 µM MG132 in the presence and absence of 250 pM TGFβ1 for 24 h. Cells were either lysed and immunoblotted for P-Smad2, Smad2 and LC3B or subjected to fluorescence microscopy. In both cell lines, the quantitation of GFP- and RFP-labelled LC3 in the western blots showed that TGFβ1 significantly decreased the GFP/RFP ratio but increased steady state LC3B-II protein levels (Fig. 5A,C,E; Figs S6A, S7A, and S8A). Quantitation of the western blots containing chloroquine treatments indicated that chloroquine significantly increased steady state LC3B-II protein levels and the GFP/RFP ratio compared to untreated A549 cells (Fig. 5A) and H1299 cells (Fig. S6A). Additionally, the combination of chloroquine and TGFβ1 significantly increased the GFP/RFP ratio with respect to the TGFβ1 treatment in both cell lines (Fig. 5A; Fig. S6A). Consistent with the western blotting results, quantifying the GFP/RFP ratio via fluorescence microscopy indicated that compared to untreated cells, TGFβ1 decreased the GFP/RFP ratio and chloroquine increased the GFP/RFP ratio in A549 cells and H1299 cells. Furthermore, the combination of chloroquine and TGFβ1 had a significantly greater GFP/RFP ratio compared to the TGFβ1 treatment in both cell lines (Fig. 5B; Fig. S6B). Having observed consistent results using chloroquine, an inhibitor of late-stage autophagy events, we next assessed spautin-1, which inhibits earlier autophagic processes.

Quantitation of the western blots containing spautin-1 treated cell suggested that the combination of spautin-1 and TGFβ1 significantly decreased steady state LC3B-II protein levels and increased the GFP/RFP ratio with respect to the TGFβ1 treatment in both cell lines (Fig. 5C; Fig. S7A). Quantifying the GFP/RFP ratio via fluorescence microscopy indicated that TGFβ1 decreased the GFP/RFP ratio, whereas spautin-1 increased the GFP/RFP ratio. Finally, the combination of spautin-1 and TGFβ1 had a significantly greater GFP/RFP ratio compared to the TGFβ1 treatment (Fig. 5D; Fig. S7B). Taken together, these results were consistent in showing that the inhibition of early or late autophagic events produced similar GFP/RFP autophagic flux ratios (Fig. 5A,C, right panels). Finally, quantitation of the western blots containing MG132 treatments revealed that MG132 significantly decreased the GFP/RFP ratio whereas it increased LC3B-II protein levels with respect to the DMSO control in A549 cells (Fig. 5E) and H1299 cells (Fig. S8A). Additionally, compared to TGFβ1 treatment, the combination of MG132 and TGFβ1 significantly decreased the GFP/RFP ratio in H1299 cells, but increased steady state LC3B-II protein levels in both cell lines (Fig. 5E; Fig. S7C). Quantifying the GFP/RFP ratio via fluorescence microscopy indicated that TGFβ1 and MG132 decreased the GFP/RFP ratio in both cell lines (Fig. 5F; Fig. S7D). Taken together, this data suggested that chloroquine and spautin-1 decreased autophagic flux whereas MG132 and TGFβ1 increased autophagic flux in the NSCLC cell lines.

**Fig. 6.**
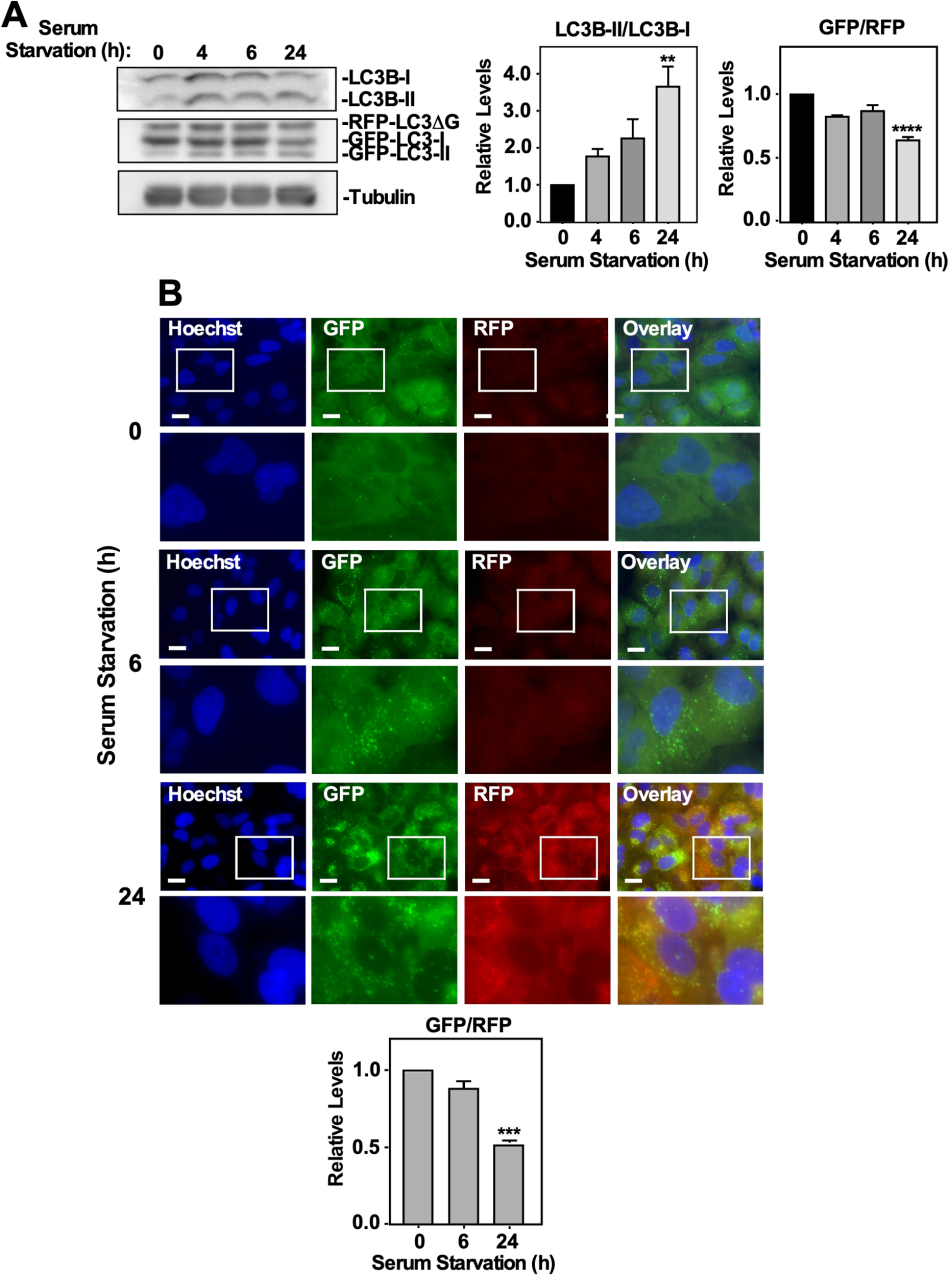
**Assessing autophagic flux via serum starvation and the effect of ATG5 and ATG7 silencing on TGFβ1-dependent autophagy in A549 cells.** (A) A549 cells stably expressing GFP-LC3-RFP-LC3ΔG were serum starved for 0, 4, 6 or 24 h, lysed, subjected to SDS-PAGE and immunoblotted with anti-LC3B and anti-tubulin antibodies. Quantitative analysis of LC3B-II/LC3B-I and GFP/RFP ratios are shown graphically to the right of representative immunoblots. The data were graphed from three independent experiments (mean±s.e.m.). Significance is indicated as **=*P*<0.01 and ****P<0.0001. (B) A549 cells stably expressing GFP-LC3-RFP-LC3ΔG were serum starved for 0, 6 or 24 h. Hoechst stain (blue) was added 10 min prior to imaging. Images were obtained with a 63x objective using an Olympus IX 81 inverted fluorescence microscope and ImageJ was used to quantify the green and red pixel intensity. The GFP/RFP ratio is shown below representative images. The data were graphed from three independent experiments (mean±s.e.m.). Significance is indicated as ***=*P*<0.001. Scale bars: 10 μm.

Since this autophagic flux probe suggested that chloroquine and spautin-1 are inhibitors of autophagy, whereas TGFβ1 and MG132 were observed to activate autophagy, we confirmed these results by assessing this autophagic flux model using starvation-induced autophagy, which has been shown to induce autophagy via an ULK-1-dependent mechanism (Wong et al., 2013). A549 cells or H1299 cells stably transfected with a GFP-LC3-RFP-LC3ΔG construct were serum starved for 0–24 h prior to being lysed and immunoblotted for LC3B and tubulin or subjected to fluorescence microscopy. In A549 cells, 24 h of serum starvation significantly increased and decreased LC3B-II/LC3B-I and GFP/RFP ratios, respectively (Fig. 6A). Quantifying the GFP/RFP fluorescence microscopy autophagic flux ratio revealed that 24 h of serum starvation significantly increased autophagy with respect to the control (Fig. 6B). In H1299 cells, 4 and 6 h of serum starvation significantly increased the LC3B-II/LC3B-I ratio whereas 4, 6 and 24 h of serum starvation significantly decreased the GFP/RFP ratio (Fig. S9A). Quantifying the GFP/RFP ratio via fluorescence microscopy revealed that 6 and 24 h of serum starvation significantly decreased the GFP/RFP ratio with respect to the control (Fig. S9B). Therefore, this data suggested that serum starvation increased autophagic flux in our NSCLC cell lines.

### Utilizing GFP-LC3-RFP-LC3ΔG transfected cells to assess the role of ATG5 and ATG7 on TGFβ1-dependent autophagy

**Fig. 7.**
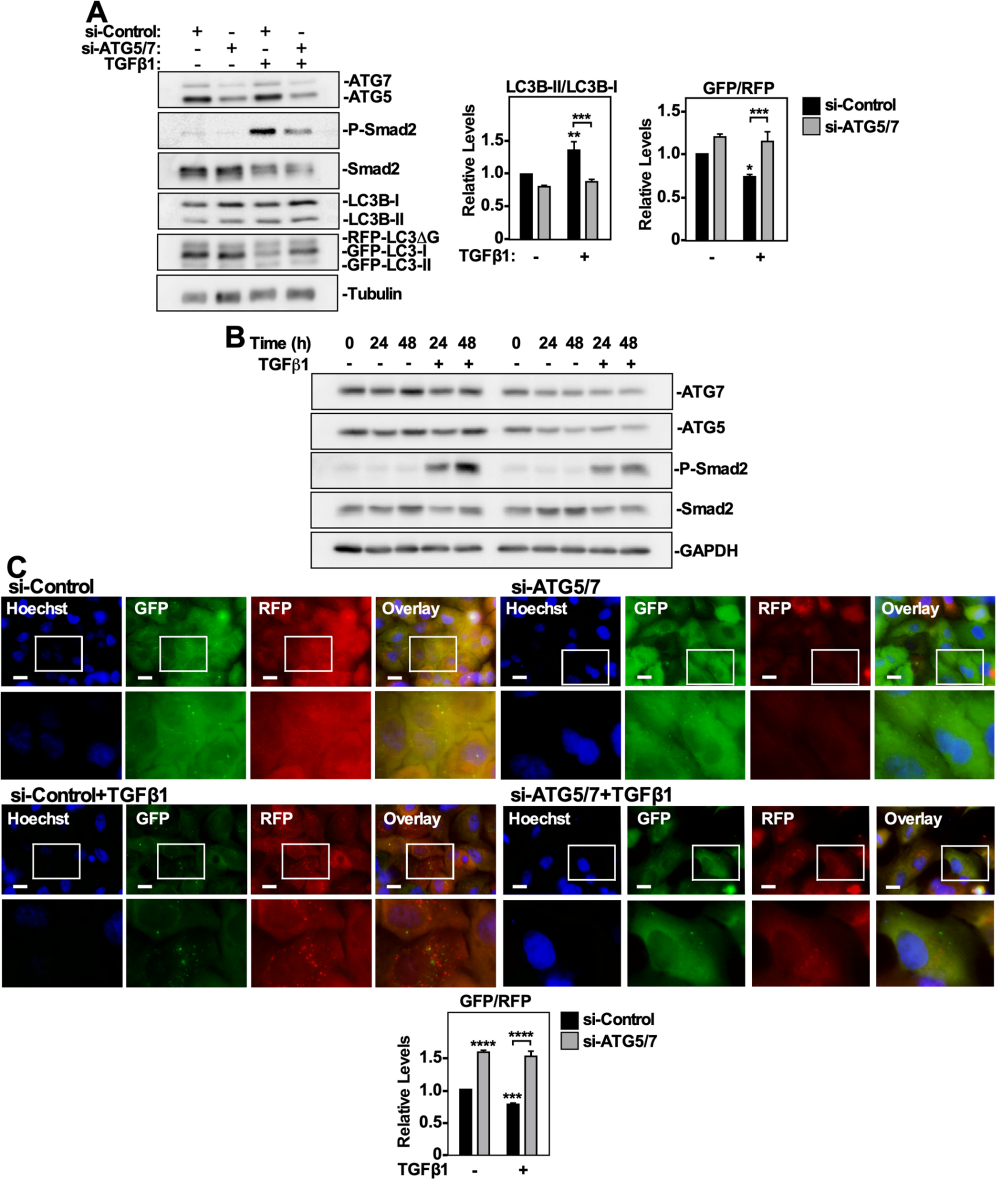
**The effect of ATG5 and ATG7 silencing on TGFβ1-dependent autophagy in A549 cells.** (A) A549 cells stably expressing GFP-LC3-RFP-LC3ΔG were transfected with control siRNA (si-Control) or siRNA targeting ATG5 and ATG7 (si-ATG5/7) were incubated for 24 h in the presence or absence of 250 pM TGFβ1. The cells were lysed, subjected to SDS-PAGE and immunoblotted with anti-ATG7, anti-ATG5, anti-P-Smad2, anti-Smad2, anti-LC3B and anti-tubulin antibodies. Quantitative analysis of LC3B-II/LC3B-I and GFP/RFP ratios are shown graphically to the right of representative immunoblots. The data were graphed from three independent experiments (mean±s.e.m.). Significance is indicated as *=*P*<0.05, **=*P*<0.01 and ***P<0.001. (B) A549 cells stably expressing GFP-LC3-RFP-LC3ΔG were incubated with 250 pM TGFβ1for 0, 24 or 48 h. Cells were then lysed and immunoblotted with anti-ATG7, anti-ATG5, anti-P-Smad2, anti-Smad2 and anti-GAPDH antibodies. (C) A549 cells stably expressing GFP-LC3-RFP-LC3ΔG were transfected with control siRNA (si-Control) or siRNA targeting ATG5 and ATG7 (si-ATG5/7) were incubated for 24 h in the presence or absence of 250 pM TGFβ1. Hoechst stain (blue) was added 10 min prior to imaging. Images were obtained with a 63x objective using an Olympus IX 81 inverted fluorescence microscope and ImageJ quantified the green and red pixel intensity. The GFP/RFP ratio is shown below representative images. The data were graphed from three independent experiments (mean±s.e.m.). Significance is indicated as ***=*P*<0.001 and ****=*P*<0.0001. Scale bars: 10 μm.

Finally, we tested the application of GFP-LC3-RFP-LC3ΔG transfected NSCLC cells to characterize the effect that silencing *ATG5* and *ATG7* (*ATG5/7*) expression had on TGFβ1-induced autophagy. A549 cells and H1299 cells stably expressing GFP-LC3-RFP-LC3ΔG were transfected with ATG5 and ATG7 siRNA (si-ATG5/7) or control siRNA (si-Control) for 24 h. The cells were then treated with 250 pM TGFβ1 for 24 h and immunoblotted for ATG7, ATG5, P-Smad2, Smad2, and LC3B. We observed that silencing ATG5 and ATG7 significantly decreased TGFβ-dependent Smad2 phosphorylation and the LC3B-II/LC3B-I ratio, while increasing the GFP/RFP ratio in A549 cells (Fig. 7A,B) and H1299 cells (Fig. S10A,B). Furthermore, fluorescence microscopy indicated that the combination of si-ATG5/7 and TGFβ1 significantly increased the GFP/RFP autophagic ratio compared to TGFβ1-treated control cells, indicating that ATG5/7 silencing decreased TGFβ-dependent autophagy. (Fig. 7C; Fig. S10C). Taken together, these data show that ATG5 and ATG7 are essential for TGFβ1-dependent autophagy in both A549 and H1299 NSCLC cells, and suggests that inhibiting autophagy impacts TGFβ signaling potential.

## DISCUSSION

We examined the utility of monitoring autophagy via *ATG* gene expression, ATG protein levels, LC3B lipidation, LC3 puncta formation and autophagosome-lysosome co-localization. Using NSCLC cells, we found that TGFβ1 had limited effects on *ATG* gene expression and altered the protein levels of a subset of autophagy-related proteins (ATG3 and ULK1). However, it increased LC3B lipidation, LC3 puncta formation and autophagosome-lysosome co-localization. Another experimental technique that we considered for this study was transmission electron microscopy (TEM). Although TEM is considered the gold standard to identify double-membrane vesicles observed during cellular autophagy (Ylä-Anttila et al., 2009; Biazik et al., 2015), we excluded it from this work because (1) TGFβ1 had already been suggested to increase the number of autophagosomes in NSCLC cells (Alizadeh et al., 2018), and (2) the use of this technique is limited for cell population based quantitation.

We utilized autophagy inhibitors (spautin-1 and chloroquine) and an autophagy activator (MG132) to expose the limitations of the previously mentioned techniques. However, in order to understand this, we must first recognize how these pharmacological agents modulate autophagy. For instance, spautin-1 antagonizes the activity of ubiquitin specific protease 10 and ubiquitin specific protease 13 that are responsible for removing ubiquitin from the BECN1 complex. As such, spautin-1 increases the proteasome-mediated degradation of the BECN1 complex, which ultimately decreases autophagosome formation and inhibits relatively early events during autophagy (Guo et al., 2020). Chloroquine is a lysosomotropic agent that accumulates in lysosomes and increases lysosomal pH to prevent lysosomal-mediated degradation. Therefore, autophagosomes accumulate because lysosomal-mediated degradation is reduced, and chloroquine is considered an inhibitor of later events in autophagy (Mauthe et al., 2018). Finally, MG132 antagonizes the catalytic subunits within the 20S core particle of the proteasome, which increases the protein load on the cell. Since proteasome and lysosome-mediated degradation are compensatory, as proteasomal activity decreases, autophagic activity increases to alleviate the excess protein load (Bao et al., 2016; Wojcik, 2013).

Although many publications have used similar experimental techniques to support that particular experimental conditions induced autophagy, repeating our experiments with known inhibitors and activators of autophagy suggested that this may not be the case. For instance, chloroquine and MG132 had little effect on ATG protein levels and increased LC3B lipidation, LC3 puncta formation and autophagosome-lysosome co-localization. The explanation for this is that many techniques rely on autophagosome structures to make interpretations of autophagy (Klionsky et al., 2016; Ylä-Anttila et al., 2009). However, many of these techniques do not monitor degradation, which is the final, and most important, stage of autophagy (Noda et al., 2009; Papadopoulos et al., 2020). Since chloroquine prevents autophagosome degradation, autophagosome accumulation will be interpreted by several techniques as increased autophagy. Other autophagy inhibitors that do not impede lysosomal degradation, such as spautin-1, are not at risk of this limitation. Furthermore, if TEM experiments were performed, based on our results and what is reported in the literature (Cechakova et al., 2019; Yang and Klionsky, 2010; Kriegenburg et al., 2018), we would expect chloroquine, MG132, serum starvation and TGFβ1 to increase the number of autophagosomes whereas spautin-1 would decrease the number of autophagosomes. Ultimately, since a known inhibitor and activator of autophagy resulted in similar outcomes, it would be inaccurate to conclude that TGFβ1 activated autophagy based on these parameters alone.

We next reviewed the literature for an experimental technique to measure autophagy that was not confounded by previously mentioned limitations. We found that assessing autophagic flux measures autophagic degradation, and as such may distinguish between autophagy activators and lysosomal inhibitors (Zhang et al., 2013). Currently, there are several autophagic flux probes available for experimentation; however, we selected to stably express the GFP-LC3-RFP-LC3ΔG construct, developed in the Mizushima laboratory (Kaizuka et al., 2016), in NSCLC cells. Using these cell lines, we determined that TGFβ1 elicited a time-dependent decrease of the GFP/RFP ratio, which suggests increased autophagy. Indeed, since GFP-LC3 undergoes autophagic degradation whereas the RFP-LC3ΔG does not, a decrease in the GFP/RFP ratio suggested that autophagic flux increased. We next validated our model using chloroquine, spautin-1, MG132 and serum starvation. As expected, the autophagy inhibitors increased the GFP/RFP ratio whereas autophagy activators decreased the GFP/RFP ratio. Based on these data, we concluded that TGFβ1 activated autophagy in NSCLC cells.

After we verified that TGFβ1 activated autophagy, we provided an example as to how we may use our GFP-LC3-RFP-LC3ΔG transfected cells to characterize the mechanism of TGFβ1-induced autophagy. We have shown that TGFβ1-induced autophagy is dependent on the presence of ATG5 and ATG7. Although this may seem straightforward because ATG5 and ATG7 are key components for phagophore elongation, ATG5/7-independent autophagy is possible (Walczak and Martens, 2013; Arakawa et al., 2017). In order to characterize TGFβ1-dependent autophagy, future experiments will aim to impede specific components of the canonical and non-canonical TGFβ1 signaling pathways and assess the impact on autophagic flux. This work will determine the exact TGFβ1 signaling pathway(s) that is/are responsible for autophagy activation so that we may aim to impede a specific branch of TGFβ1 signaling rather than the entire pathway. This is important because TGFβ1 is essential to cell survival and plays an anti-tumorigenic role in most cells. As such, the application of this model may identify specific targets of the TGFβ pathway that are directly responsible for autophagy activation so that we may specifically hinder them in cancer to limit off-target and non-specific effects.

## MATERIALS AND METHODS

### Antibodies and reagents

Primary antibodies were purchased from the following vendors: anti-BECN1 (Cell Signaling Technology, 3738S), anti-GAPDH (Cell Signaling Technology, 2118S), anti-tubulin (Cell Signaling Technology, 2144S), anti-LC3B (Cell Signaling Technology, 9236S), anti-ULK1 (Cell Signaling Technology, 8054S) anti-P-Smad2 (Cell Signaling Technology, 3108L), anti-Smad2/3 (BD Transduction laboratories, 562586), anti-ATG3 (Cell Signaling Technology, 3415S), anti-ATG5 (Cell Signaling Technology, 12994S), anti-ATG7 (Cell Signaling Technology, 8558S), anti-ATG9A (Novus Biologicals, NB110-56893), anti-ATG16L1 (Cell Signaling Technology, 8089S) and anti-ATG12 (Cell Signaling Technology, 4180S). Secondary antibodies used for western blot analysis were as follows: horseradish-peroxidase-conjugated goat anti-rabbit-IgG (Thermo Fisher Scientific, 31460) and goat anti-mouse-IgG (Thermo Fisher Scientific, 31430). Human Ambion small interfering (si)RNA constructs were purchased from Thermo Fisher Scientific (si-ATG7, si-ATG5 and si-Control with catalog numbers 4392420, 4392420 and 4457289, respectively). For fluorescence microscopy, LysoTracker Deep Red (Invitrogen, L12492) and Hoechst stain (Invitrogen, H3569) labelled lysosomes and nuclei, respectively. The pharmacological agents that modulate autophagy were spautin-1 (spautin-1) (Sigma-Aldrich, SML0440), chloroquine (chloroquine) (Acquired from the Shepherd lab, London, Canada) and MG132 (Sigma-Aldrich, M7449).

### Cell culture and transfections

H1299 cells and A549 NSCLC cells were purchased from ATCC and cultured in Roswell Park Memorial Institute (RPMI; Corning, 10-043-CVR) and Kaighn's Modification of Hams F-12 (F-12K; Corning, 10-025-CV) media, respectively. Both cell lines were passaged using 0.25% trypsin EDTA (Sigma-Aldrich, T2605), centrifuged at 1000×***g*** for 2 min and resuspended in fresh media supplemented with 10% FBS. A humidified tissue incubator cultured the cells at 37°C under 5% CO_2_. Cells were treated with 250 pM TGFβ1, 50 µM chloroquine, 10 µM spautin-1, 10 µM MG132 in media supplemented with 10% FBS. Transient siRNA knockdowns in H1299 cells and A549 cells were performed using optimem media (Thermo Fisher Scientific, 22600134) and Lipofectamine RNAiMAX (Thermo Fisher Scientific, 13778150) as per the manufacturer’s protocol. Stable GFP-LC3-RFP-LC3ΔG expressing cells were generated using a cDNA pMRX-IP-GFP-LC3-RFP-LC3ΔG vector (Addgene, 84573) and PolyJet transfection reagent (Froggabio, Toronto, ON, Canada). Transfected cells were isolated using 1 µg/ml puromycin (Thermo Fisher Scientific, A1113802) in growth media supplemented with 10% FBS.

### Microarray data analysis

Expression of autophagy specific genes were analyzed from our previously published microarray dataset of untreated and TGFβ-treated A549 cells (49; NCBI Gene Expression Omnibus website, GEO; GSE26241).

### Immunoblotting

Protein isolation was achieved using a 1× TNTE lysis buffer containing 50 mM Tris pH 7.5, 150 mM sodium chloride, 1 mM EDTA, 0.5% Triton X-100, 1 mg/mL pepstatin, 50 μM PMSF, 2.5 mM sodium fluoride, and 10 mM sodium pyrophosphate phosphatase inhibitor. Cell lysates were then centrifuged at 21000 ***g*** at 4°C for 12 min. Protein concentration was determined using the DC^TM^ protein assay (Bio-Rad, Hercules, CA, USA) and a Victor 3 V Multi-Detection Microplate Reader (PerkinElmer, Waltham, MA, USA). Prior to immunoblotting, 8x loading buffer [30% glycerol, 10% 1.5 M Tris (pH 6.8), 1.2% SDS, 0.018% bromophenol blue, and 15% β-mercaptoethanol] was added to the protein lysates. Protein lysates mixed with loading buffer were utilized for sodium dodecyl sulfate polyacrylamide gel electrophoresis (SDS-PAGE). Each well of the polyacrylamide gel received approximately 50 μg of protein, which was run at a constant 120 volts for 100 min. Following a standard wet transfer protocol, proteins were transferred onto a nitrocellulose membrane using a constant 100 volts for 80 min. Nitrocellulose membranes were blocked with 5% skim milk for 1 h, rocking at room temperature. Primary antibodies were incubated overnight with the nitrocellulose membranes, rocking at 4°C. On the following day, nitrocellulose membranes were incubated with the appropriate HRP-conjugated secondary antibody for 1 h at room temperature. Enhanced chemiluminescent substrate (Bio-Rad, 1705060) was added 5 min prior to visualizing using a Versa-doc Imager (Bio-Rad). Finally, QuantityOne^®^ 1-D Analysis software (Bio-Rad) was used to analyze the relative intensity of protein bands.

### MTT assay

A549 cells and H1299 cells were treated with increasing concentrations of chloroquine, spautin-1 or MG132 for 24 and 48 h in a 96 well plate. After the incubation period, the cells were subject to an MTT assay (Sigma-Aldrich, 11465007001) as per the manufacturers’ protocol. Briefly, we added 10 µl of the MTT labelling reagent to each well and left it in a humidified tissue incubator at 37°C under 5% CO_2_ for 4 h. Next, we added 100 µl of the solubilization solution to each well and placed it in a humidified tissue incubator overnight. The next day a Victor 3V Multi-Detection Microplate Reader measured the absorbance of the 550 nm and 690 nm wavelengths. The values for each wavelength were subtracted by the blank (cell null) treatment wavelength values. The 550–690 nm absorbances for the no treatment cells were standardized to 100% viability and all treatments were relative to the no treatment control.

### Assessing autophagosome and lysosome co-localization

A549 cells stably expressing GFP-LC3 were treated with pharmacological modulators of autophagy in the presence and absence of TGFβ1 for 24 h. LysoTracker Deep Red labelled lysosomes and Hoechst stain labelled the nucleus 2 h and 10 min prior to imaging, respectively. Using a 60x objective of an Olympus IX 81 inverted fluorescence microscope (Olympus, Canada), we imaged the Hoechst stain, GFP-LC3 and LysoTracker Deep Red. Co-localization was observed by the appearance of yellow puncta, which suggested that the GFP-LC3 and lysosomes were in close proximity. The colocalization plug-in of ImageJ version 2.0 quantified each image. Each data point represents quantitation from ≥100 cells from each condition.

### Autophagic flux assay

Autophagic flux was measured using A549 cells and H1299 cells that were transfected with a cDNA pMRX-IP-GFP-LC3-RFP-LC3ΔG vector developed by the Mizushima laboratory (30; Addgene). After the transfected cells express this vector, they produce two forms of LC3: LC3 conjugated to green fluorescent protein (GFP-LC3) and a mutant LC3 with a C-terminal glycine deletion conjugated to red fluorescent protein (RFP-LC3ΔG). The LC3ΔG cannot be incorporated into the autophagosome membrane, and thus as autophagy occurs the GFP-LC3 is degraded whereas the RFP-LC3ΔG remains immune to autophagic degradation. (Kaizuka et al., 2016) Immunoblotting using LC3B specific antibodies could distinguish the RFP-LC3ΔG, GFP-LC3-I and GFP-LC3-II bands, which are quantified using QuantityOne^®^ 1-D Analysis software to determine the GFP/RFP ratio. Furthermore, using a 60x objective of an Olympus IX 81 inverted fluorescence microscope, we imaged the green and red channels. The GFP/RFP ratio was determined by ImageJ version 2.0, which quantified the average pixel intensity for green and red channels.

### Statistical analysis

One-way ANOVA followed by Dunnett's multiple comparisons test, a two-way/three-way ANOVA followed by either Tukey's or Sidak's multiple comparison tests and Student's *t*-tests were used to evaluate the significance of the results. Statistical analyses were performed using GraphPad Prism Software 8.1 and *P*-values <0.05 were considered to be statistically significant.

## Supplementary Material

Supplementary information
