# Supplementary material for: Assessing methods to quantitatively validate TGFβ-dependent autophagy
Source: Biol Open. 2020 Nov 26;9(11):bio055103. doi: 10.1242/bio.055103 (PMC7710024; doi:10.1242/bio.055103)
Supplement: Supplementary information [file biolopen-9-055103-s1.pdf]

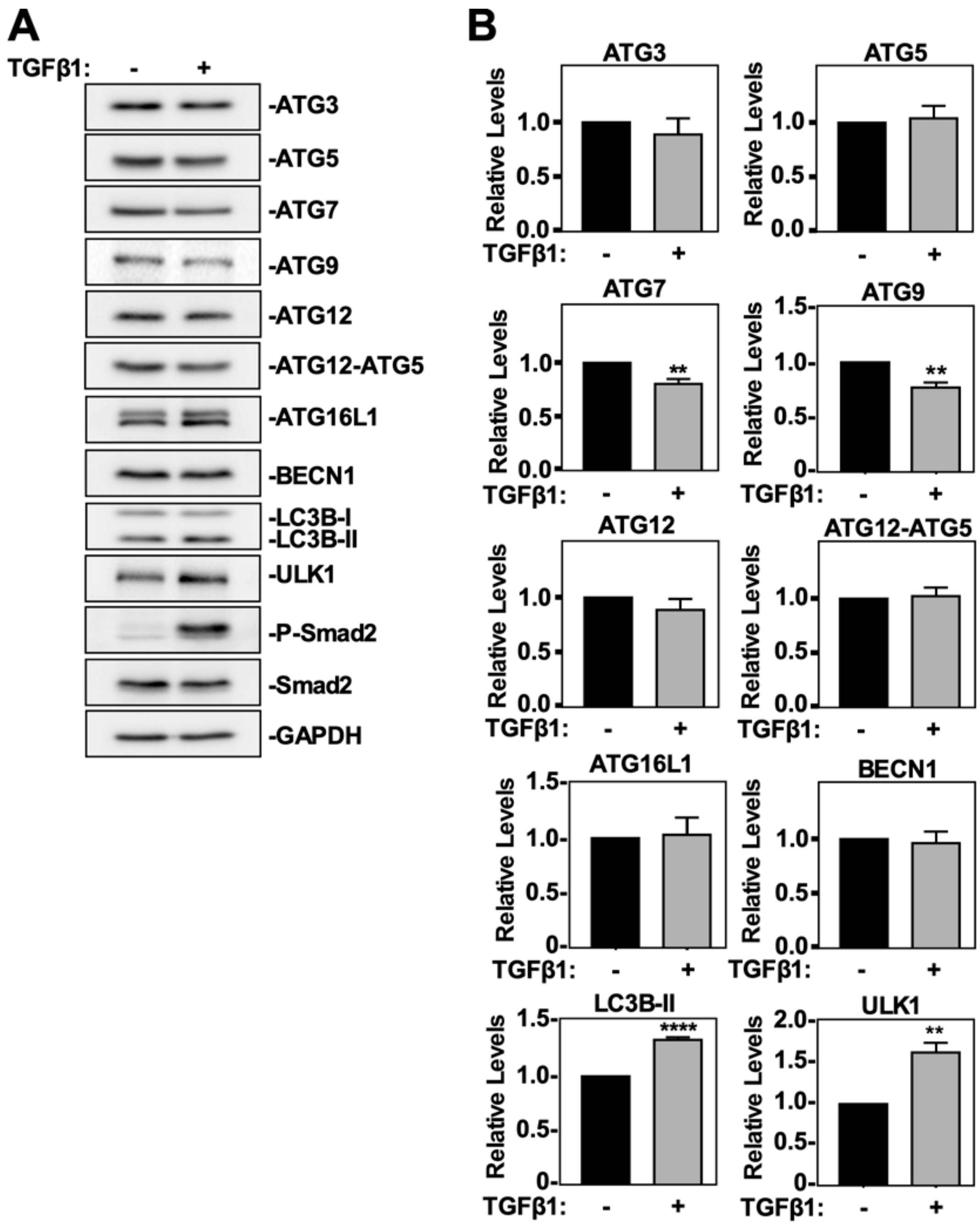

Supplemental Figure 1. Trelford and Di Guglielmo

Biology Open • Supplementary information

Figure S1. The effect of TGFβ1 on ATG protein levels and LC3B lipidation in H1299 cells.
A) H1299 cells were treated with 250 pM TGFβ1 for 24 hours. Cells were lysed and subjected to SDS-PAGE and immunoblotting using anti-ATG3, anti-ATG5, anti-ATG7, anti-ATG9, anti-ATG12, anti-ATG12-ATG5 complex, anti-ATG16L1, anti-BECN1, anti-ULK1, anti-LC3B, anti-P-Smad2, anti-Smad2 and anti-GAPDH (loading control) antibodies.
B) Phosphoimaging analysis of steady state levels of ATG7, ATG5, ATG3, ATG12-ATG5, GAPDH, BECN1, ATG12 and LC3B were quantitated and graphed (n=3± SEM). Significance is indicated as *=P<0.05, **P<0.01 and ****=P<0.0001.

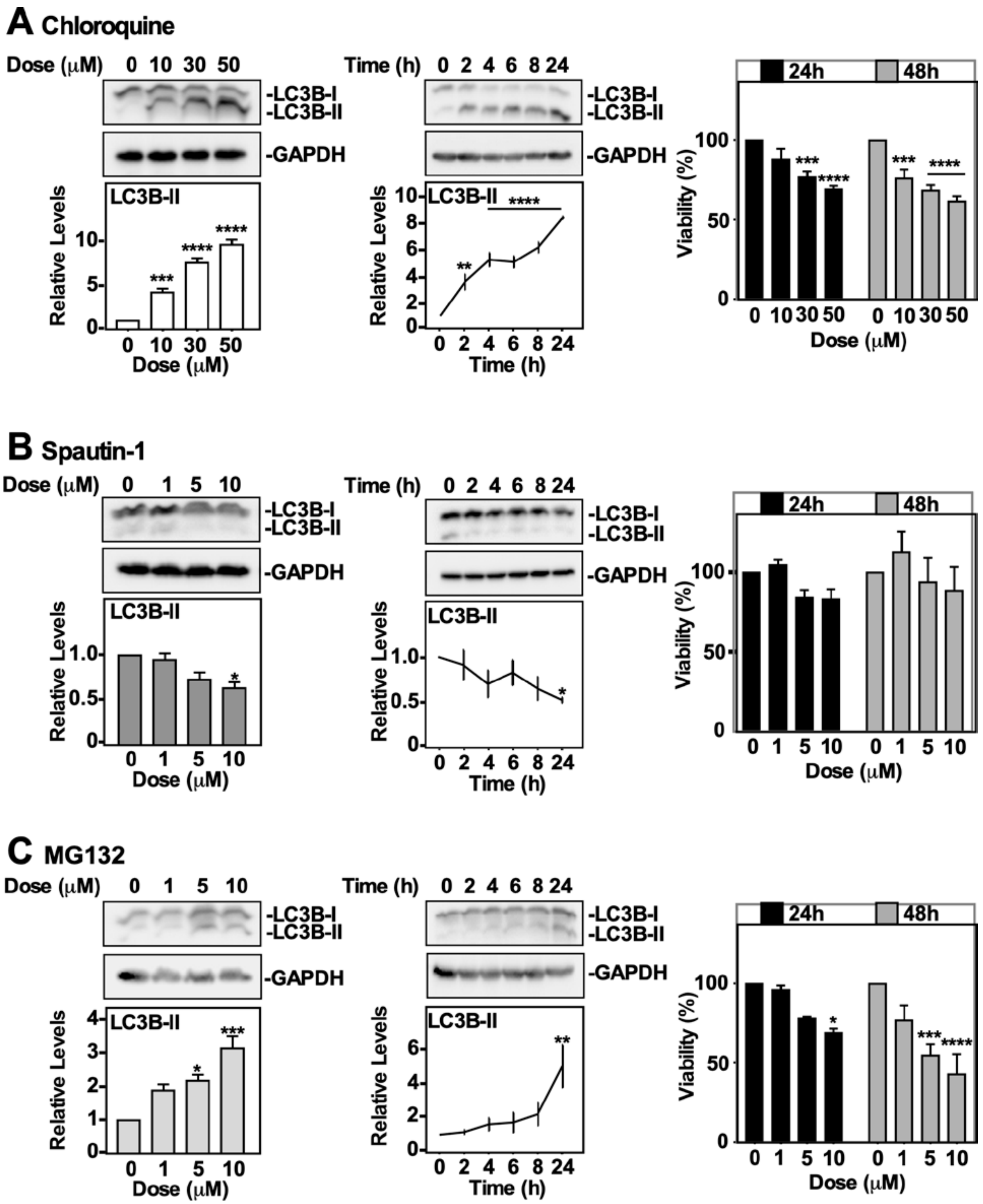

Supplemental Figure 2. Trelford and Di Guglielmo

Biology Open • Supplementary information

Figure S2. The effects of chloroquine, spautin-1 and MG132 on LC3 lipidation and viability of A549 cells.

A) A549 cells were treated with 0-50 µM chloroquine for 24 hours (left panel) or A549 cells were treated with 50 µM chloroquine for 0, 2, 4, 6, 8 or 24 hours (middle panel). Cells were lysed and subjected to SDS-PAGE and immunoblotting using anti-GAPDH and anti-LC3B antibodies. Quantitative analysis of steady state LC3B levels are shown graphically below representative immunoblots. A549 cells were treated with 0-50 µM chloroquine for 24 or 48 hours and subjected to an MTT assay (right panel). The data were graphed from 4 independent experiments (mean ± SEM). Significance is indicated as **P<0.01, ***=P<0.001 and ****=P<0.0001.

B) A549 cells were treated with 0-10 µM spautin-1 for 24 hours (left panel) or A549 cells were treated with 10 µM spautin-1 for 0, 2, 4, 6, 8 or 24 hours (middle panel). Cells were lysed and subjected to SDS-PAGE and immunoblotting using anti-GAPDH and anti-LC3B antibodies. Quantitative analysis of steady state LC3B levels are shown graphically below representative immunoblots. A549 cells were treated with 0-10 µM spautin-1 for 24 or 48 hours and subjected to an MTT assay (right panel). The data were graphed from 4 independent experiments (mean ± SEM). Significance is indicated as *=P<0.05.

C) A549 cells were treated with 0-10 µM MG132 for 24 hours (left panel) or A549 cells were treated with 10 µM MG132 for 0, 2, 4, 6, 8 or 24 hours (middle panel). Cells were lysed and subjected to SDS-PAGE and immunoblotting using anti-GAPDH and anti-LC3B antibodies. Quantitative analysis of steady state LC3B levels are shown graphically below representative immunoblots. A549 cells were treated with 0-10 µM MG132 for 24 or 48 hours and subjected to an MTT assay (right panel). The data were graphed from 4 independent experiments (mean ± SEM). Significance is indicated as *=P<0.05, **P<0.01, ***=P<0.001 and ****=P<0.0001.

Biology Open • Supplementary information

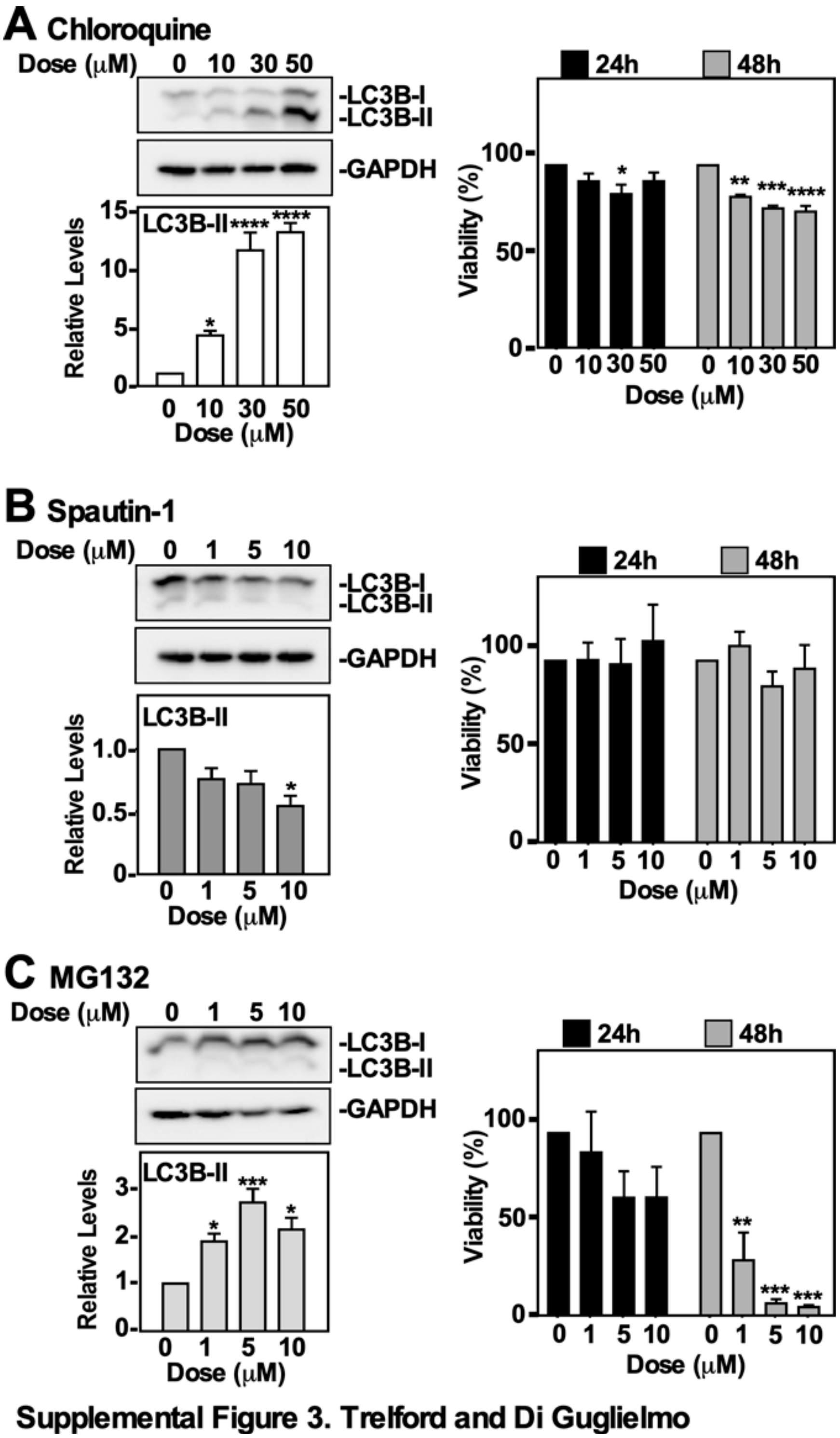

Figure S3. The effects of chloroquine, spautin-1 and MG132 on LC3 lipidation and viability of H1299 cells.

A) H1299 cells were treated with 0-50 μM chloroquine for 24 hours (left panel). Cells were lysed and subjected to SDS-PAGE and immunoblotting using anti-GAPDH and anti-LC3B antibodies.

Biology Open • Supplementary information

Phosphoimaging analysis of steady state LC3B levels are shown graphically below representative immunoblots. H1299 cells were treated with 0-50 μM chloroquine for 24 or 48 hours and subjected to an MTT assay (right panel). The data were graphed from 4 independent experiments (mean ± SEM). Significance is indicated as *=P<0.05, **P<0.01, ***=P<0.001 and ****=P<0.0001.

B) H1299 cells were treated with 0-10 μM spautin-1 for 24 hours (left panel). Cells were lysed and subjected to SDS-PAGE and immunoblotting using anti-GAPDH and anti-LC3B antibodies. Phosphoimaging analysis of steady state LC3B levels are shown graphically below representative immunoblots. H1299 cells were treated with 0-10 μM spautin-1 for 24 or 48 hours and subjected to an MTT assay (right panel). The data were graphed from 4 independent experiments (mean ± SEM). Significance is indicated as *=P<0.05.

C) H1299 cells were treated with 0-10 μM MG132 for 24 hours (left panel). Cells were lysed and subjected to SDS-PAGE and immunoblotting using anti-GAPDH and anti-LC3B antibodies. Phosphoimaging analysis of steady state LC3B levels are shown graphically below representative immunoblots. H1299 cells were treated with 0-10 μM MG132 for 24 or 48 hours and subjected to an MTT assay (right panel). The data were graphed from 4 independent experiments (mean ± SEM). Significance is indicated as *=P<0.05, **P<0.01 and ***=P<0.001.

Biology Open • Supplementary information

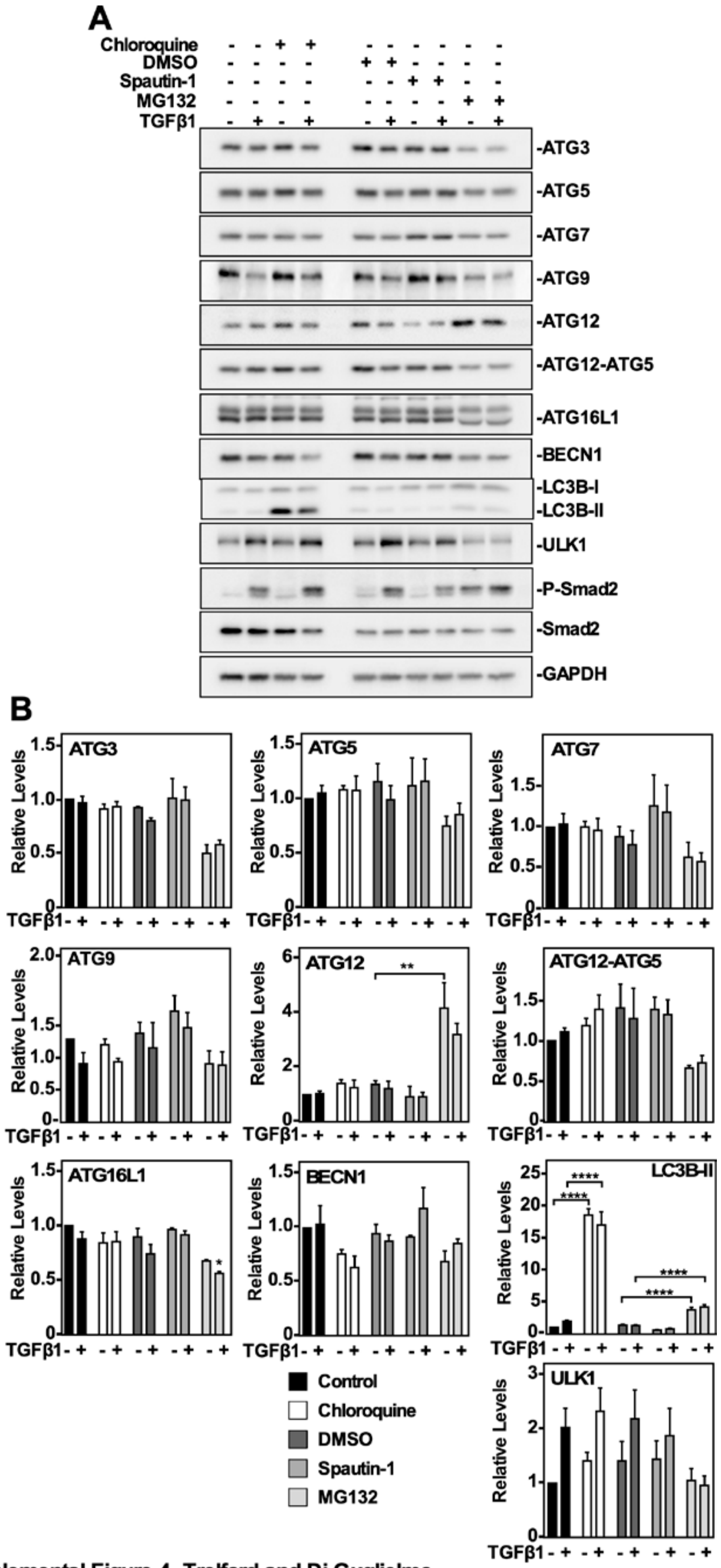

Supplemental Figure 4. Trelford and Di Guglielmo

Figure S4. The effect of chloroquine, spautin-1 and MG132 on ATG protein levels in H1299 cells.

A) H1299 cells were treated with 50 µM chloroquine, 10 µM spautin-1 or 5 µM MG132 in the presence or absence of 250 pM TGFβ1 for 24 hours. Cells were lysed and subjected to SDS-PAGE and immunoblotting using anti-ATG3, anti-ATG5, anti-ATG7, anti-ATG9, anti-ATG12, anti-ATG12-ATG5 complex, anti-ATG16L1, anti-BECN1, anti-ULK1, anti-LC3B, anti-P-Smad2, anti-Smad2 and anti-GAPDH (loading control) antibodies.

B) Phosphoimaging analysis of steady state ATG7 levels, ATG5 levels, ATG3 levels, ATG12-ATG5 levels, GAPDH levels, BECN1 levels, ATG12 levels, and LC3B levels were graphed. The data were graphed from 3 independent experiments (mean ± SEM). Significance is indicated as *=P<0.05, **P<0.01 and ****=P<0.0001.

Biology Open • Supplementary information

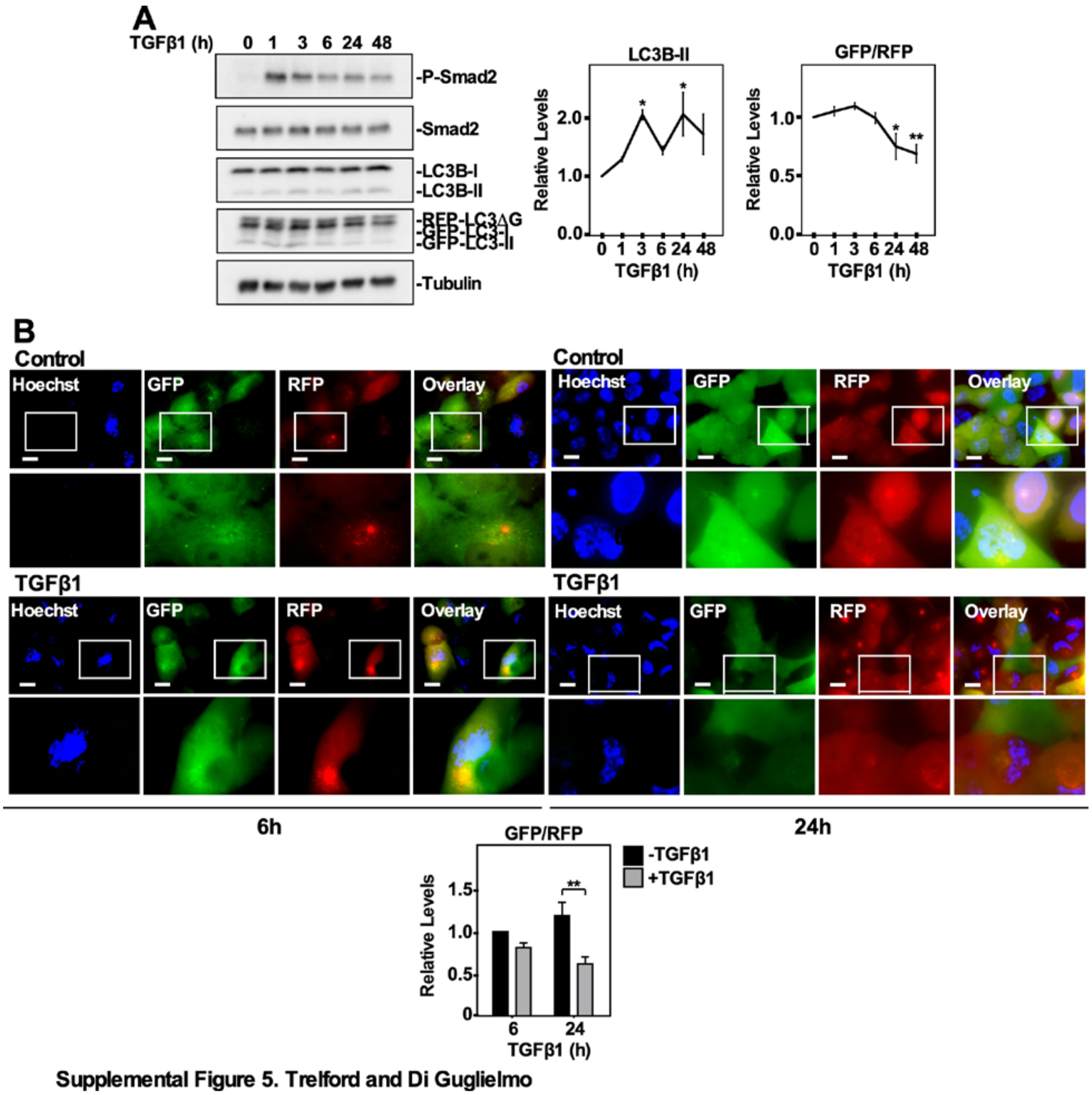

Figure S5. Using a pMRX-IP-GFP-LC3-RFP-LC3ΔG probe to assess TGFβ1-dependent autophagy in H1299 cells.

A) H1299 cells stably expressing GFP-LC3-RFP-LC3ΔG were treated with 250 pM TGFβ1 for 0-48 hours, lysed, subjected to SDS-PAGE and immunoblotted with anti-P-Smad2, anti-Smad2, anti-LC3B, anti-GAPDH and anti-tubulin antibodies. Phosphoimaging analysis of steady state LC3B-II levels and the GFP/RFP ratio are shown graphically to the right of representative immunoblots. The data were graphed from 3 independent experiments (mean ± SEM). Significance is indicated as *=P<0.05 and **P<0.01.

B) H1299 cells stably expressing GFP-LC3-RFP-LC3ΔG were treated with 250 pM TGFβ1for 6 or 24 hours. Hoechst stain (blue) was added 10 minutes prior to imaging. Images were obtained

Biology Open • Supplementary information

with a 63x objective using an Olympus IX 81 inverted fluorescence microscope and Image J quantified the green and red pixel intensity. The GFP/RFP ratio is shown to the right of representative images. The data were graphed from 3 independent experiments (mean ± SEM). Significance is indicated as **=P<0.01. Scale bars = 10 μm.

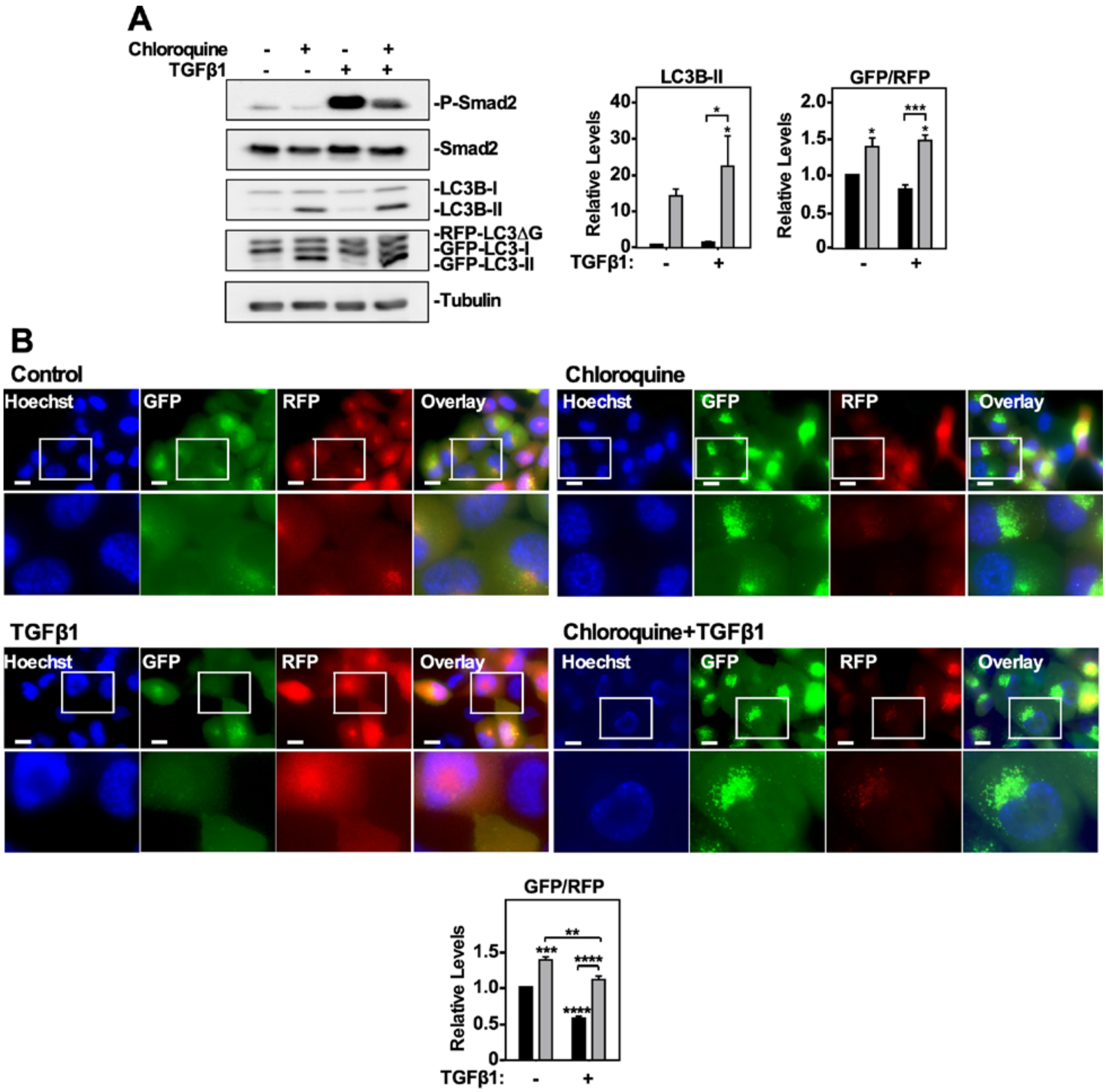

Supplemental Figure 6. Trelford and Di Guglielmo

Figure S6. The effect of chloroquine on autophagic flux in H1299 cells.

A) H1299 cells stably expressing GFP-LC3-RFP-LC3ΔG were treated with 50 μM chloroquine in the presence and absence of 250 pM TGFβ1 for 24 hours, lysed, subjected to SDS-PAGE and immunoblotted with anti-P-Smad2, anti-Smad2, anti-LC3B, anti-GAPDH and anti-tubulin antibodies. Phosphoimaging analysis of steady state LC3B-II levels and the GFP/RFP ratio are shown graphically to the right of representative immunoblots. The data were graphed from 3 independent experiments (mean ± SEM). Significance is indicated as *=P<0.05 and ***P<0.001.

B) H1299 cells stably expressing GFP-LC3-RFP-LC3ΔG were treated with 50 μM chloroquine in the presence and absence of 250 pM TGFβ1for 6 or 24 hours. Hoechst stain (blue) was added 10

minutes prior to imaging. Images were obtained with a 63x objective using an Olympus IX 81 inverted fluorescence microscope and Image J quantified the green and red pixel intensity. The GFP/RFP ratio is shown below representative images. The data were graphed from 3 independent experiments (mean ± SEM). Significance is indicated as **P<0.01, ***=P<0.001 and ****=P<0.0001. Scale bars = 10 μm.

Biology Open • Supplementary information

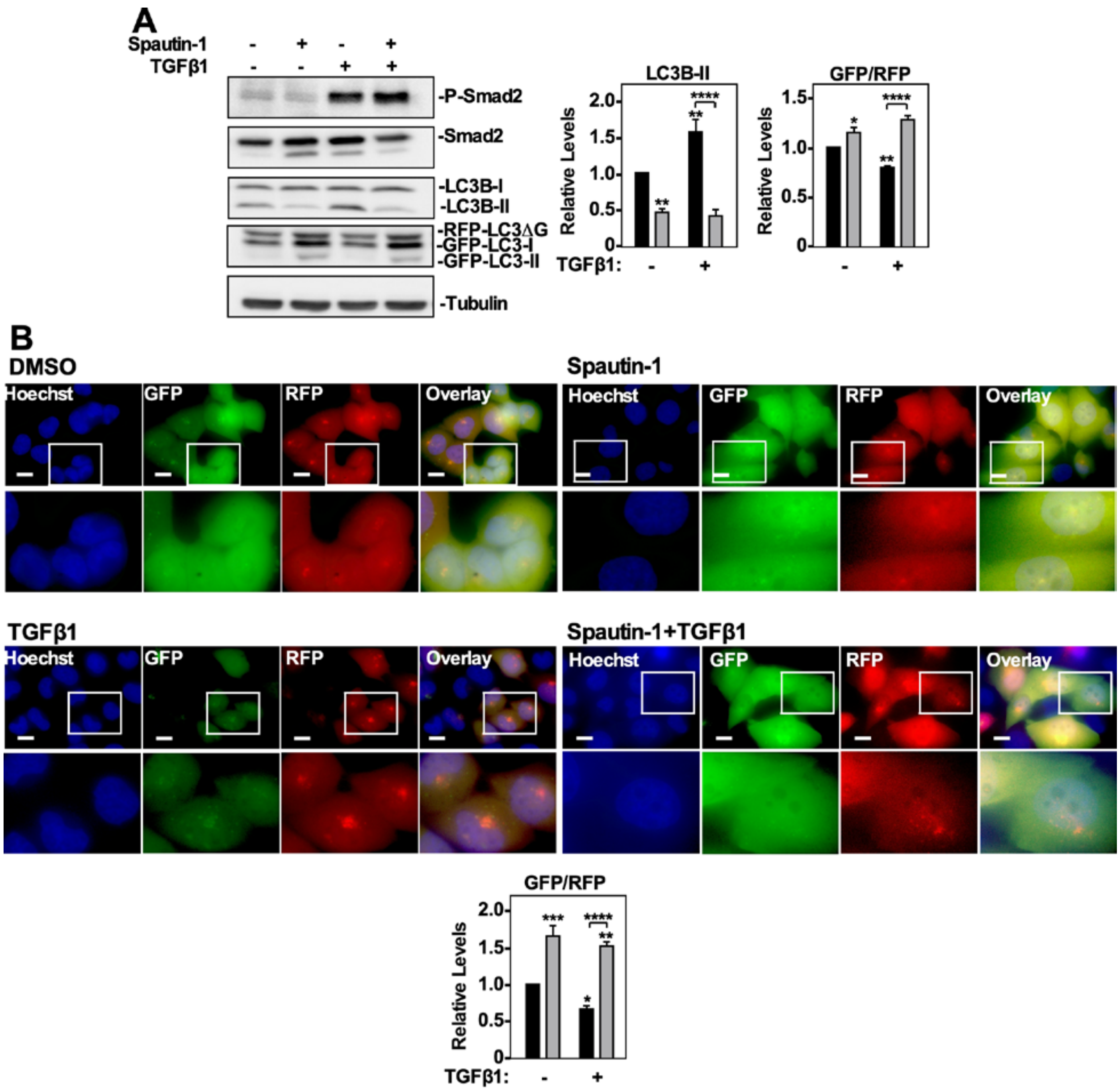

Supplemental Figure 7. Trelford and Di Guglielmo

Figure S7. The effect of spautin-1 on autophagic flux in H1299 cells.

A) H1299 cells stably expressing GFP-LC3-RFP-LC3ΔG were treated with 10 µM spautin-1 in the presence and absence of 250 pM TGFβ1 for 24 hours, lysed, subjected to SDS-PAGE and immunoblotted with anti-P-Smad2, anti-Smad2, anti-LC3B, anti-GAPDH and anti-tubulin antibodies. Phosphoimaging analysis of steady state LC3B-II levels and the GFP/RFP ratio are shown graphically to the right of representative immunoblots. The data were graphed from 3 independent experiments (mean ± SEM). Significance is indicated as *=P<0.05, **=P<0.01 and ****=P<0.0001.

B) H1299 cells stably expressing GFP-LC3-RFP-LC3ΔG were treated with 10 µM spautin-1 in the presence and absence of 250 pM TGFβ1 for 6 or 24 hours. Hoechst stain (blue) was added 10

minutes prior to imaging. Images were obtained with a 63x objective using an Olympus IX 81 inverted fluorescence microscope and Image J quantified the green and red pixel intensity. The GFP/RFP ratio is shown below representative images. The data were graphed from 3 independent experiments (mean ± SEM). Significance is indicated as *=$P<0.05$, **$P<0.01$, ***$P<0.001$ and ****=$P<0.0001$. Scale bars = 10 µm.

Biology Open • Supplementary information

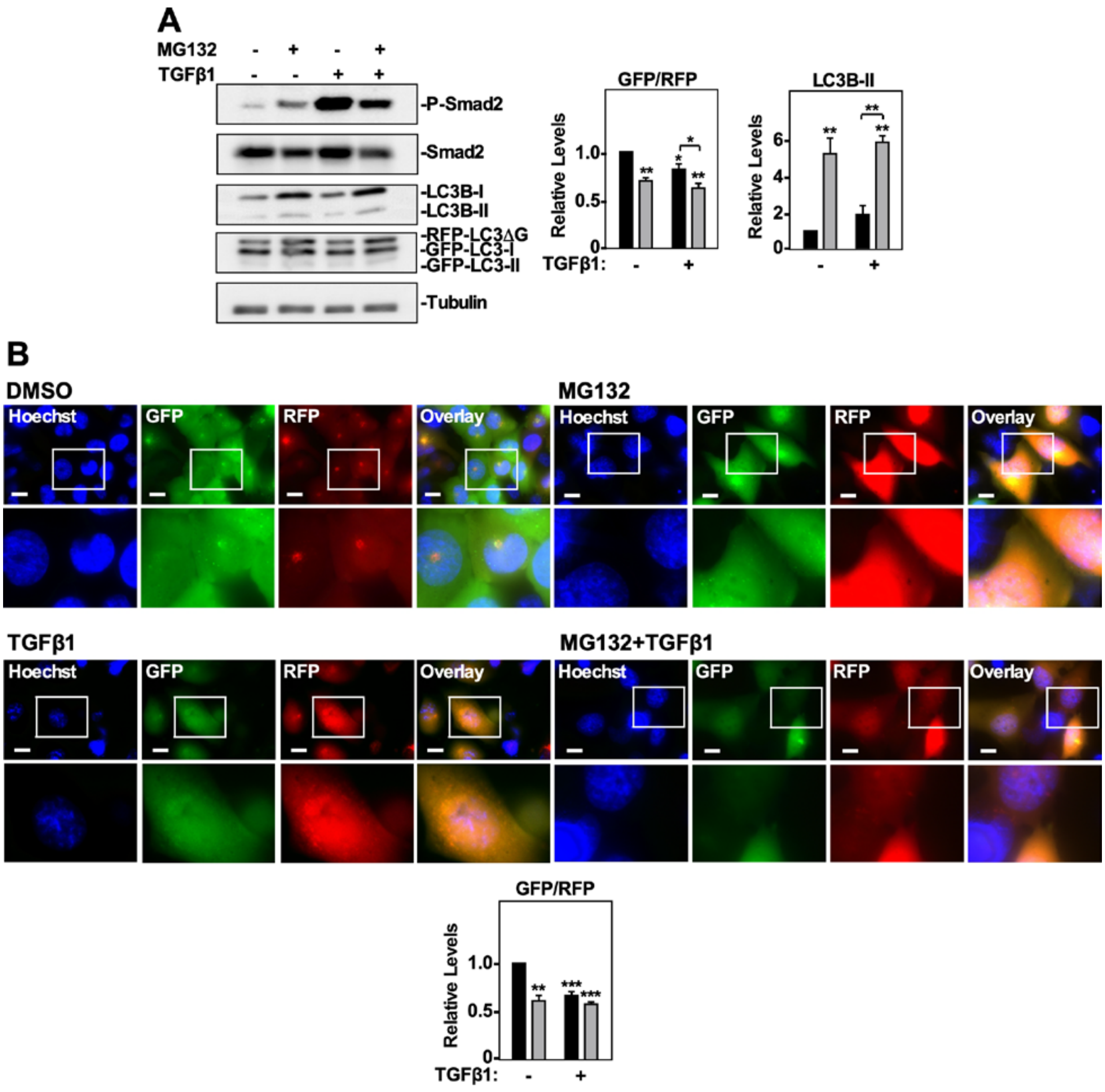

Supplemental Figure 8. Trelford and Di Guglielmo

Figure S8. The effect of MG132 on autophagic flux in H1299 cells.

A) H1299 cells stably expressing GFP-LC3-RFP-LC3ΔG were treated with 5 µM MG132 in the presence and absence of 250 pM TGFβ1 for 24 hours, lysed, subjected to SDS-PAGE and immunoblotted with anti-P-Smad2, anti-Smad2, anti-LC3B, anti-GAPDH and anti-tubulin antibodies. Phosphoimaging analysis of steady state LC3B-II levels and the GFP/RFP ratio are shown graphically to the right of representative immunoblots. The data were graphed from 3 independent experiments (mean ± SEM). Significance is indicated as *=P<0.05, **P<0.01, ***P<0.001 and ****=P<0.0001.

B) H1299 cells stably expressing GFP-LC3-RFP-LC3ΔG were treated with 5 µM MG132 in the presence and absence of 250 pM TGFβ1 for 6 or 24 hours. Hoechst stain (blue) was added 10

Biology Open • Supplementary information

minutes prior to imaging. Images were obtained with a 63x objective using an Olympus IX 81 inverted fluorescence microscope and Image J quantified the green and red pixel intensity. The GFP/RFP ratio is shown below representative images. The data were graphed from 3 independent experiments (mean ± SEM). Significance is indicated as $^{**}P<0.01$ and $^{***}P<0.001$. Scale bars = 10 μm.

Biology Open • Supplementary information

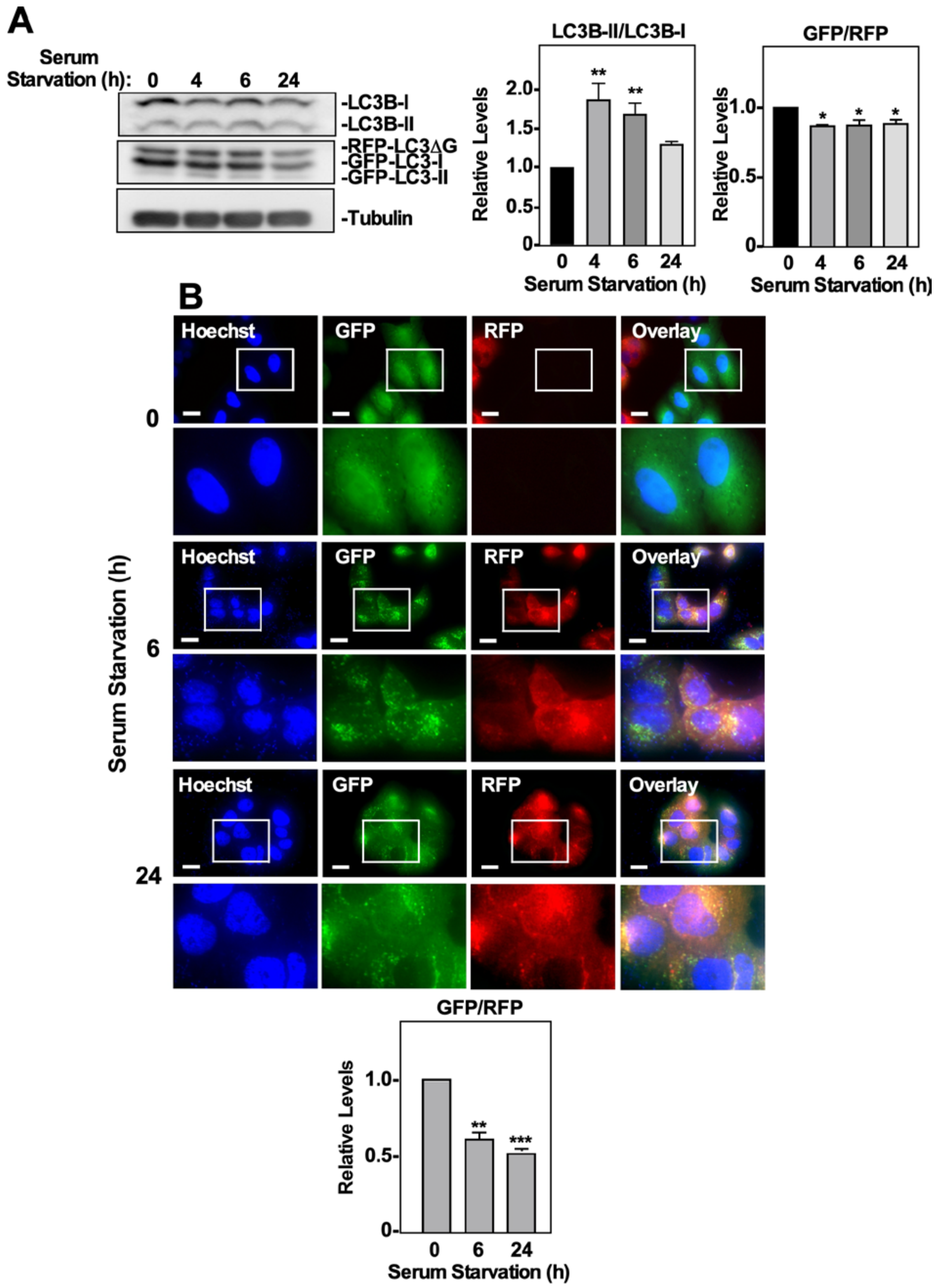

Supplemental Figure 9. Trelford and Di Guglielmo

Biology Open • Supplementary information

Figure S9. Using a GFP-LC3-RFP-LC3ΔG probe to assess serum starvation induced autophagy in H1299 cells.

A) H1299 cells stably expressing GFP-LC3-RFP-LC3ΔG were serum starved for 0, 4, 6 or 24 hours, lysed, subjected to SDS-PAGE and immunoblotted with anti-LC3B and anti-tubulin antibodies. Phosphoimaging analysis of LC3B-II/LC3B-I and GFP/RFP ratios are shown graphically to the right of representative immunoblots. The data were graphed from 3 independent experiments (mean ± SEM). Significance is indicated as *=P<0.05 and **=P<0.01.

B) H1299 cells stably expressing GFP-LC3-RFP-LC3ΔG were serum starved for 0, 6 or 24 hours. Hoechst stain (blue) was added 10 minutes prior to imaging. Images were obtained with a 63x objective using an Olympus IX 81 inverted fluorescence microscope and Image J quantified the green and red pixel intensity. The GFP/RFP ratio is shown below representative images. The data were graphed from 3 independent experiments (mean ± SEM). Significance is indicated as **=P<0.01 and ***=P<0.001. Scale bars = 10 μm.

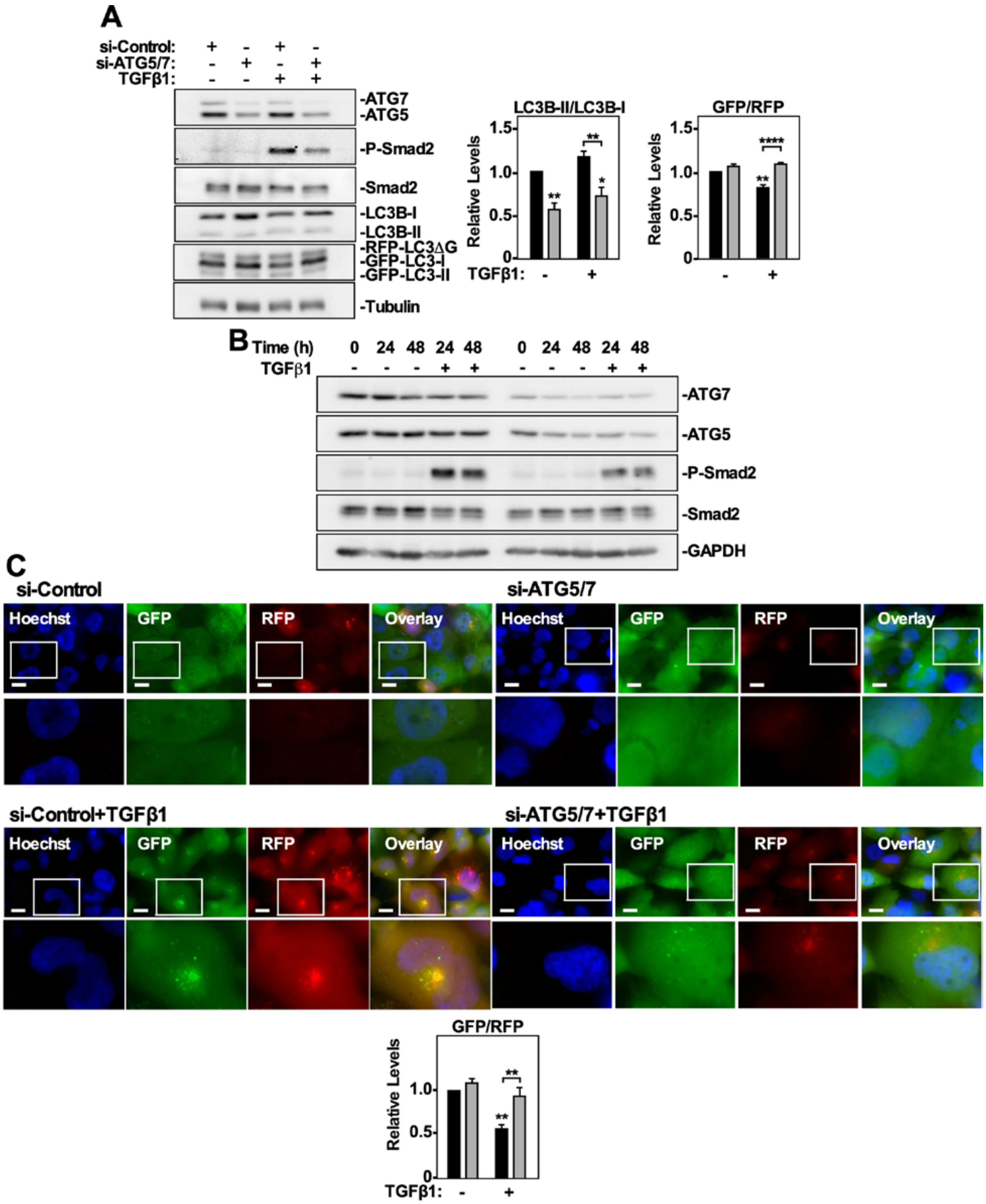

Supplemental Figure 10. Trelford and Di Guglielmo

Figure S10. The effect of ATG5 and ATG7 silencing on TGFβ1-dependent autophagy in H1299 cells.

Biology Open • Supplementary information

A) H1299 cells stably expressing GFP-LC3-RFP-LC3ΔG were transfected with control siRNA (si-Control) or siRNA targeting ATG5 and ATG7 (si-ATG5/7) were incubated for 24 hours in the presence or absence of 250 pM TGFβ1. The cells were lysed, subjected to SDS-PAGE and immunoblotted with anti-ATG7, anti-ATG5, anti-P-Smad2, anti-Smad2, anti-LC3B, anti-GAPDH and anti-tubulin antibodies. Phosphoimaging analysis of LC3B-II/LC3B-I and GFP/RFP ratios are shown graphically to the right of representative immunoblots. The data were graphed from 3 independent experiments (mean ± SEM). Significance is indicated as *=P<0.05, **=P<0.01 and ****P<0.0001.

B) H1299 cells stably expressing GFP-LC3-RFP-LC3ΔG were incubated with 250 pM TGFβ1for 0, 24 or 48h. Cells were then lysed and immunoblotted with anti-ATG7, anti-ATG5, anti-P-Smad2, anti-Smad2 and anti-GAPDH antibodies.

C) H1299 cells stably expressing GFP-LC3-RFP-LC3ΔG were transfected with control siRNA (si-Control) or siRNA targeting ATG5 and ATG7 (si-ATG5/7) were incubated for 24 hours in the presence or absence of 250 pM TGFβ1. Hoechst stain (blue) was added 10 minutes prior to imaging. Images were obtained with a 63x objective using an Olympus IX 81 inverted fluorescence microscope and Image J quantified the green and red pixel intensity. The GFP/RFP ratio is shown below representative images. The data were graphed from 3 independent experiments (mean ± SEM). Significance is indicated as ***=P<0.001 and **=P<0.01. Scale bars = 10 μm.

Biology Open • Supplementary information
